# Molecular Dynamics Simulations of Forced Unbending of Integrin α_V_β_3_

**DOI:** 10.1371/journal.pcbi.1001086

**Published:** 2011-02-17

**Authors:** Wei Chen, Jizhong Lou, Jen Hsin, Klaus Schulten, Stephen C. Harvey, Cheng Zhu

**Affiliations:** 1 Woodruff School of Mechanical Engineering, Georgia Institute of Technology, Atlanta, Georgia, United States of America; 2 Institute for Bioengineering and Bioscience, Georgia Institute of Technology, Atlanta, Georgia, United States of America; 3 Department of Physics and Beckman Institute, University of Illinois at Urbana-Champaign, Urbana, Illinois, United States of America; 4 School of Biology, Georgia Institute of Technology, Atlanta, Georgia, United States of America; 5 Coulter Department of Biomedical Engineering, Georgia Institute of Technology, Atlanta, Georgia, United States of America; University of Leeds, United Kingdom

## Abstract

Integrins may undergo large conformational changes during activation, but the dynamic processes and pathways remain poorly understood. We used molecular dynamics to simulate forced unbending of a complete integrin α_V_β_3_ ectodomain in both unliganded and liganded forms. Pulling the head of the integrin readily induced changes in the integrin from a bent to an extended conformation. Pulling at a cyclic RGD ligand bound to the integrin head also extended the integrin, suggesting that force can activate integrins. Interactions at the interfaces between the hybrid and β tail domains and between the hybrid and epidermal growth factor 4 domains formed the major energy barrier along the unbending pathway, which could be overcome spontaneously in ∼1 µs to yield a partially-extended conformation that tended to rebend. By comparison, a fully-extended conformation was stable. A newly-formed coordination between the α_V_ Asp457 and the α-genu metal ion might contribute to the stability of the fully-extended conformation. These results reveal the dynamic processes and pathways of integrin conformational changes with atomic details and provide new insights into the structural mechanisms of integrin activation.

## Author Summary

Proteins can regulate their functions via conformational changes. One example is integrins, which are transmembrane receptors mediating cell-cell and cell-matrix adhesions. Inactive integrins may assume a bent conformation with low affinities for ligands unable to support adhesions. Intracellular or extracellular stimuli induce large scale changes from the bent to an extended conformation, resulting in active integrins with high affinities for ligands to mediate strong adhesions. We used molecular dynamics simulations to reveal the dynamics and pathways of integrin unbending in atomic details. Critical interactions in this process were identified. This study not only sheds light on the structural mechanisms of integrin activation, but also exemplifies allosteric regulations of protein functions.

## Introduction

**Figure 1:**
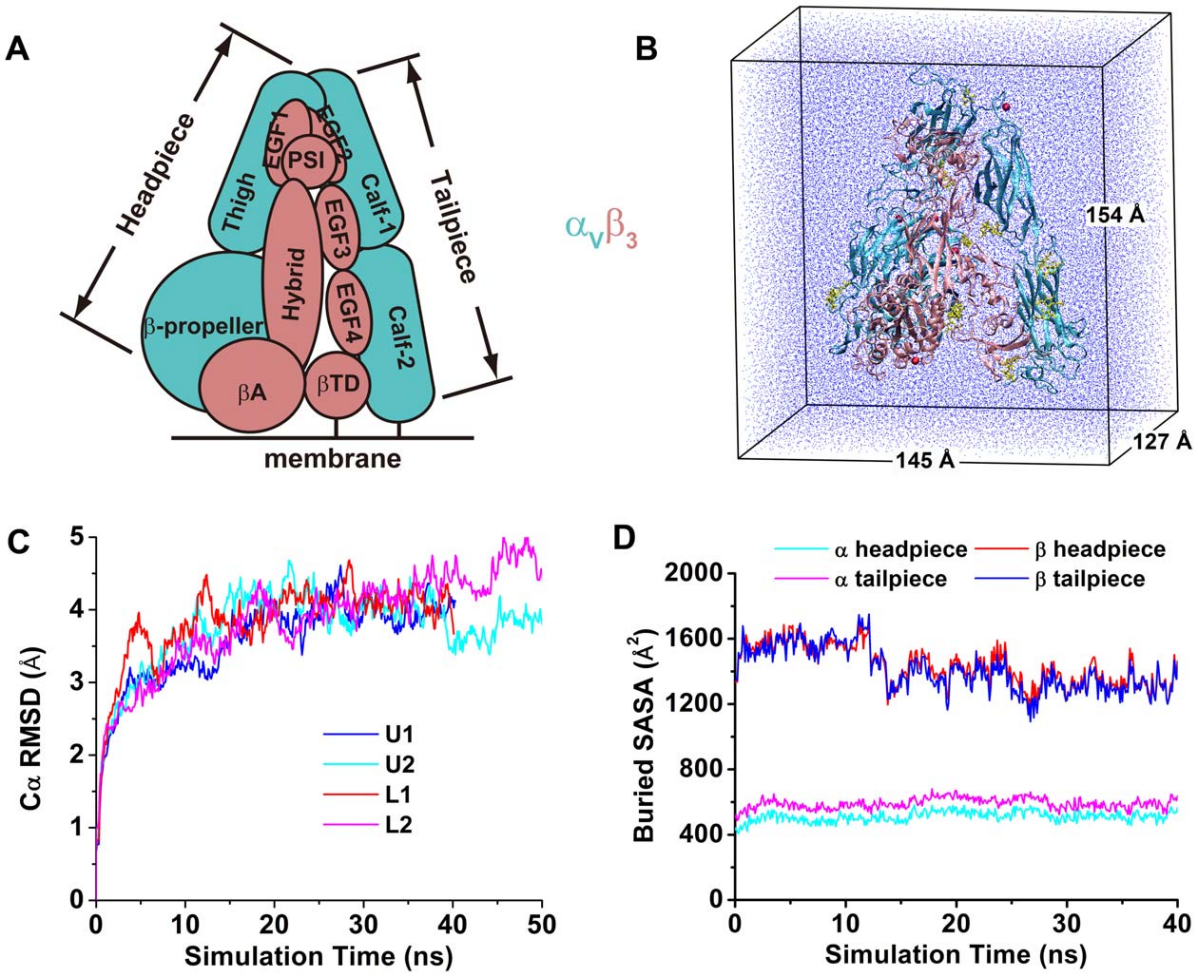
System equilibration. **A**. Schematic of an extracellular portion of integrin α_V_β_3_ showing the various domains of the α_V_ (cyan) and β_3_ (pink) subunits. **B**. Structure of U1 in a water box used for equilibration with the integrin subunits shown in the same color scheme as in panel A. Yellow sticks represent sugars attached on the protein. Red spheres represent divalent metal ions bound to the protein. Blue dots represent water molecules. The same color codes and representations are used in all figures and videos. **C**. Cα RMSDs of the four simulated structures during equilibration. **D**. Buried SASAs between the headpiece and tailpiece of U1 during equilibration.

Integrins are αβ heterodimeric transmembrane receptors for cell-cell and cell-extracellular matrix adhesions [1]. The overall shape of an integrin ectodomain is that of a large head supported by two long legs [2], [3]. The head of αA (or αI) domain-lacking integrins, including the integrin α_V_β_3_ studied here, consists of the β-propeller domain of the α subunit and the βA (or βI) domain of the β subunit (Fig. 1A). The two legs contain the thigh domain and the calf-1 and -2 domains of the α subunit and the hybrid, plexin-semaphorin-integrin (PSI), epidermal growth factor (EGF) 1–4 domains and the β tail domain (βTD) of the β subunit.

Under physiological conditions, integrins may assume an inactive state with low affinities for ligands. Upon activation by extracellular or intracellular stimuli, they may change conformations and bind ligands with high affinities and transduce signals across the plasma membrane [1]. A bent conformation, where the legs are bent at the knees, or genua, between the thigh and calf-1 domains of the α subunit and between the EGF1 and EGF2 domains of the β subunit to allow the N-terminal headpiece (from the N-termini to the knees) to contact the C-terminal tailpiece (from the knees to the C-termini) (Fig. 1A), was observed in all published crystal structures of complete integrin ectodomains, including α_V_β_3_ [4]–[7], α_IIb_β_3_ [8], and α_X_β_2_ [9]. In contrast to the “closed” headpieces observed in these bent integrins, crystal structures of an integrin α_IIb_β_3_ headpiece adopt an open conformation with the hybrid domain swinging out ∼60° [10]. Electron microscopy (EM) studies have observed different global conformations for integrins under different conditions, including a bent conformation, an extended conformation with a closed headpiece, and an extended conformation with an open headpiece [11]–[13]. A switchblade model has been proposed such that integrins undergo large global conformational changes from bent to extended during activation [11], [14]. Alternatively, a deadbolt model suggests much smaller conformational changes such that the CD loop (the β hairpin loop between the β strands C and D) of the βTD acts as a regulable deadbolt, the relief of which unlocks the βA domain from the inactive state [15].

As transmembrane mechanical links between intracellular and extracellular environments [1], integrins are often subjected to tensile forces applied externally at the head, where ligands bind, or internally at the cytoplasmic tail, where the cytoskeleton attaches. It seems intuitive that such pulling force may straighten a bent integrin and even induce other forms of conformational changes, leading to integrin activation. Indeed, force has been shown to strengthen integrin-mediated adhesion [16], [17] and prolong integrin-ligand bond lifetimes [18], [19].

Computational approaches, especially molecular dynamics (MD), have been used to study force-induced integrin conformational changes. These included steered MD (SMD) to simulate the opening of the α_V_β_3_ headpiece [20], [21], targeted MD (TMD) and normal mode analysis (NMA) to study the conformational change of the β_3_ headpiece [22], TMD to simulate the hybrid domain swing-out in the headpieces of both integrin α_V_β_3_ and α_IIb_β_3_ [23], NMA to investigate the unbending of the ectodomain of integrin α_V_β_3_ that lacks the PSI, EGF1, and EGF2 domains at the knee region [24], and SMD to simulate the conformational change between the closed and open legs on an extended model of the complete ectodomain of integrin α_IIb_β_3_ [8].

Extending from these studies, we performed all-atom, explicit-solvent MD simulations for the complete ectodomain of integrin α_V_β_3_ in both unliganded and liganded forms. Constant-velocity pulling of the head gradually unbent the integrin without major distortions of individual domains. Constant-force pulling induced a rapid transition from the bent to extended conformation after a waiting time, which increased exponentially with decreasing force to extrapolate to ∼1 µs at zero force. Two groups of interactions between the headpiece and tailpiece of the β_3_ subunit have been identified as the major barrier along the unbending pathway. A partially-extended conformation was observed to spontaneously bend back. A fully-extended conformation remained straight, which might be stabilized at least partly by a new coordination between Asp457 of the α_V_ subunit and the metal ion at the α-genu. Pulling the bound ligand also unbent the integrin, suggesting that force can activate integrins.

## Results

### Equilibration of integrin α_V_β_3_ systems

The present work includes a total of >600 ns all-atom, explicit-solvent MD simulations for four complete integrin α_V_β_3_ ectodomains (Table S1): two unliganded (U1 and U2) and two liganded forms with a cyclic RGD peptide (L1 and L2). During equilibration in a small water box (Fig. 1B), the root-mean-square deviations (RMSDs) of all Cα atoms (Cα RMSDs) first increased and then leveled off at ∼4 Å (Fig. 1C), indicating that equilibrium had been reached. All four equilibrated structures assumed a bent conformation with many contacts between the headpiece (α_V_ residues 1–600 and β_3_ residues 1–472) and tailpiece (α_V_ residues 601–956 and β_3_ residues 473–690). The calculated buried solvent-accessible surface areas (SASAs) between the headpiece and tailpiece indicate much more extensive contacts of the β_3_ subunit than the α_V_ subunit (Fig. 1D). The major barrier to unbending is therefore expected to come from the β_3_ subunit.

### Unbending of unliganded integrin α_V_β_3_ by constant-velocity SMD

**Figure 2:**
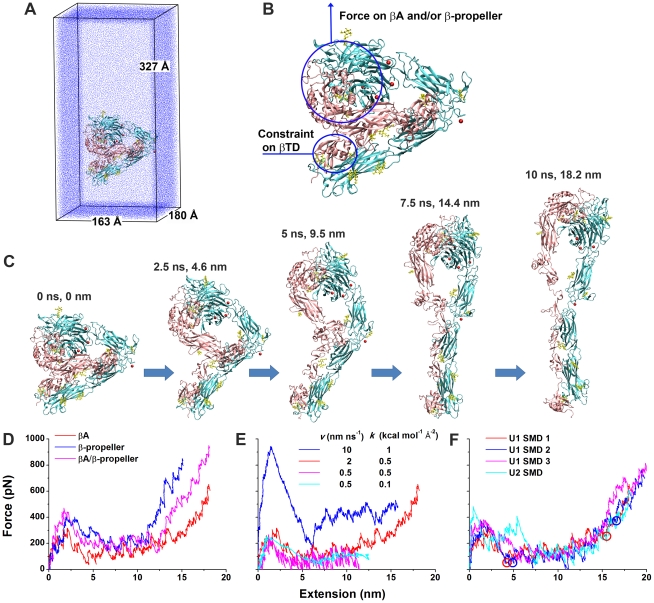
Forced unbending of unliganded integrin α_V_β_3_. **A**. U1 in the enlarged water box for unbending simulations. **B**. Illustration of force application on the head and constraint on the βTD in the SMD simulations of U1 and U2. **C**. Snapshots of a representative unbending process (U1 SMD 1) taken at indicated times and extensions. **D**. Force-extension curves in the constant-velocity SMD simulations of U1 by pulling the βA and/or β-propeller domains with a 2 nm ns^−1^ pulling speed and a 0.5 kcal mol^−1^ Å^−2^ spring constant. **E**. Force-extension curves in the constant-velocity SMD simulations of U1 by pulling the βA domain with indicated pulling speeds and spring constants. **F**. Force-extension curves for three constant-velocity SMD simulations of U1 and one constant-velocity SMD simulation of U2 with a 2 nm ns^−1^ pulling speed and a 0.5 kcal mol^−1^ Å^−2^ spring constant. Red and blue circles indicate respective structures along the unbending pathways from the trajectories of the U1 SMD 1 & 2 that were selected as starting structures for free MD simulations. The left two represent partially-extended structures and the right two represent fully-extended structures. The red curves in panels D–F are all for the U1 SMD 1.

We used constant-velocity SMD simulations [25] to accelerate integrin unbending. The enlarged systems with bigger water boxes to accommodate unbending (Fig. 2A) were first equilibrated using 4–8 ns free dynamics. A force was then applied to the head of the unliganded integrin α_V_β_3_ (U1 or U2), where ligand-binding sites are, by a spring moving at a constant velocity while the βTD was constrained to mimic the membrane anchorage (Fig. 2B). The α-leg was not constrained to allow separation of two legs, should it occur. Regardless of the initial pulling directions, force became aligned along the direction from the constraint location to the pulling location after the integrin underwent a rigid-body rotation. Remarkably, pulling readily caused gradual unbending of integrin α_V_β_3_ without major distortions of its individual domains, resulting in a fully-extended conformation with a closed headpiece and closed legs (Fig. 2C and Videos S1 & S2).

Setting the pulling speed *v* = 2 nm ns^−1^ and the spring constant *k* = 0.5 kcal mol^−1^ Å^−2^, we varied the pulling direction by loading force at different locations: βA, β-propeller, or both. Interestingly, regardless of where force was loaded, a major force peak occurred at ∼2 nm extensions (defined as the increase of distance between the constraint and pulling locations) (Fig. 2D). Setting the pulling direction by pulling the βA domain, we next varied the pulling speed from 0.5 to 10 nm ns^−1^ and the spring constant from 0.1 to 1 kcal mol^−1^ Å^−2^ (Fig. 2E). At *v* = 10 nm ns^−1^ and *k* = 1 kcal mol^−1^ Å^−2^, the pulling force rapidly increased to ∼1,000 pN at the peak level, indicating a large viscous resistant to high speed extension. At *v* = 0.5 nm ns^−1^, the force fluctuations with *k* = 0.1 kcal mol^−1^ Å^−2^ were much smaller than those with *k* = 0.5 kcal mol^−1^ Å^−2^, as expected. Importantly, a major force peak was observed at similar extensions regardless of the pulling parameters. After the major peak, force dropped to a much lower level until the integrin was straightened. These results suggest that the unbending pathway was unchanged for the different pulling parameters tested. In the following simulations, we applied force to the βA domain and chose a medium pulling speed of 2 nm ns^−1^ and a medium spring constant of 0.5 kcal mol^−1^ Å^−2^ unless otherwise indicated.

To assess the robustness of the results, we repeated constant-velocity SMD simulations three times (U1 SMD 1–3) with different equilibrated U1 structures and observed well-aligned force-extension curves with force peaks of comparable heights and at similar extensions (Fig. 2F). We also performed one constant-velocity SMD simulation for an equilibrated U2 structure (U2 SMD) and observed two major force peaks at the same extension range where the single force peaks were seen in the U1 simulations (Fig. 2F).

**Figure 3:**
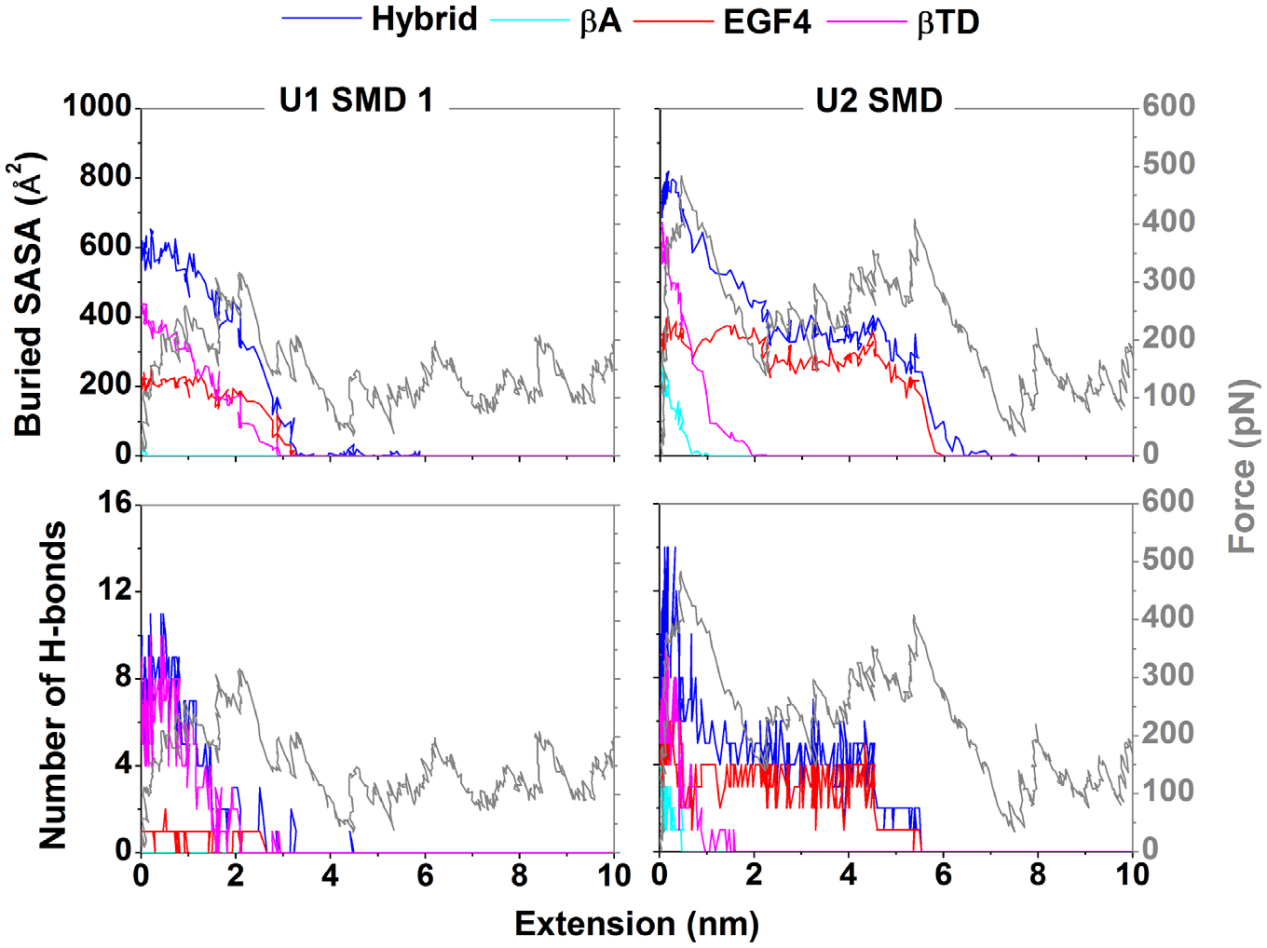
Changes in headpiece-tailpiece interactions near the major force peaks. Buried SASAs (upper row, left ordinate) and numbers of H-bonds (lower row, left ordinate) of the hybrid (blue), βA (cyan), EGF4 (red), and βTD (magenta) domains as well as pulling force (gray, both rows, right ordinate) were plotted vs. extensions for the U1 SMD 1 (left column) and U2 SMD (right column). Some of the curves were obscured due to overlapping.

To elucidate the cause of the major force peak(s), we analyzed the buried SASAs and hydrogen bonds (H-bonds) between the headpiece and tailpiece for different integrin domains (Fig. 3). The decrease in the buried SASAs of different domains during integrin unbending revealed the disruption of interdomain interactions as the originally buried surfaces were exposed. The drops of the buried SASAs of the hybrid, βTD, and EGF4 domains were found to coincide with the major force peak (Figs. 3 and S1), identifying the interactions at the hybrid/βTD and hybrid/EGF4 interfaces as the major barrier to unbending. Interactions between the βA domain and βTD also contributed to the major force peak for U2, but these interactions were broken spontaneously during the equilibration of U1 and thus not seen in the U1 SMD simulations (cyan curves in Figs. 3 and S1). In the U1 SMD 1 & 2, interactions at the two interfaces were broken at the same time. However, in the U1 SMD 3 and the U2 SMD, interactions at the hybrid/βTD interface were broken before those at the hybrid/EGF4 interface, giving rise to a small bump after the major force peak in the U1 SMD 3 and splitting the major force peak into two in the U2 SMD (gray curves in Figs. 3 and S1).

**Figure 4:**
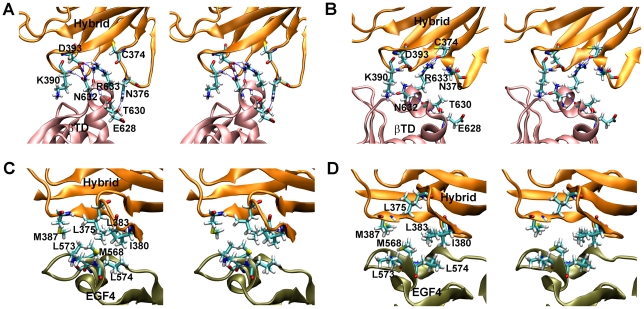
Interactions at hybrid/βTD and hybrid/EGF4 interfaces. **A & B**. Stereoviews of the post-equilibrated structures of U1 (**A**) and U2 (**B**) at the hybrid/βTD interface. **C & D**. Stereoviews of the post-equilibrated structures of U1 (**C**) and U2 (**D**) at the hybrid/EGF4 interface. Critical residues are shown as sticks. H-bonds are indicated by dashed lines. The hybrid, βTD, and EGF4 domains are colored in orange, pink, and tan, respectively.

The hybrid/βTD interface was bound mainly by polar interactions, including a salt bridge between Asp393 and Arg633 (Figs. 4 A & B) as well as H-bonds whose number decreased from 8 to 0 as the simulations passed the major force peak (Figs. 3 and S1). In contrast, the hybrid/EGF4 interface was bound mainly by hydrophobic interactions, involving several nonpolar residues (Leu375, Ile380, Leu383, Met387, Met568, Leu573, and Leu574) (Figs. 4 C & D). In addition to the hydrophobic interactions, several H-bonds were observed at the hybrid/EGF4 interface for U2 (Fig. S2).

**Figure 5:**
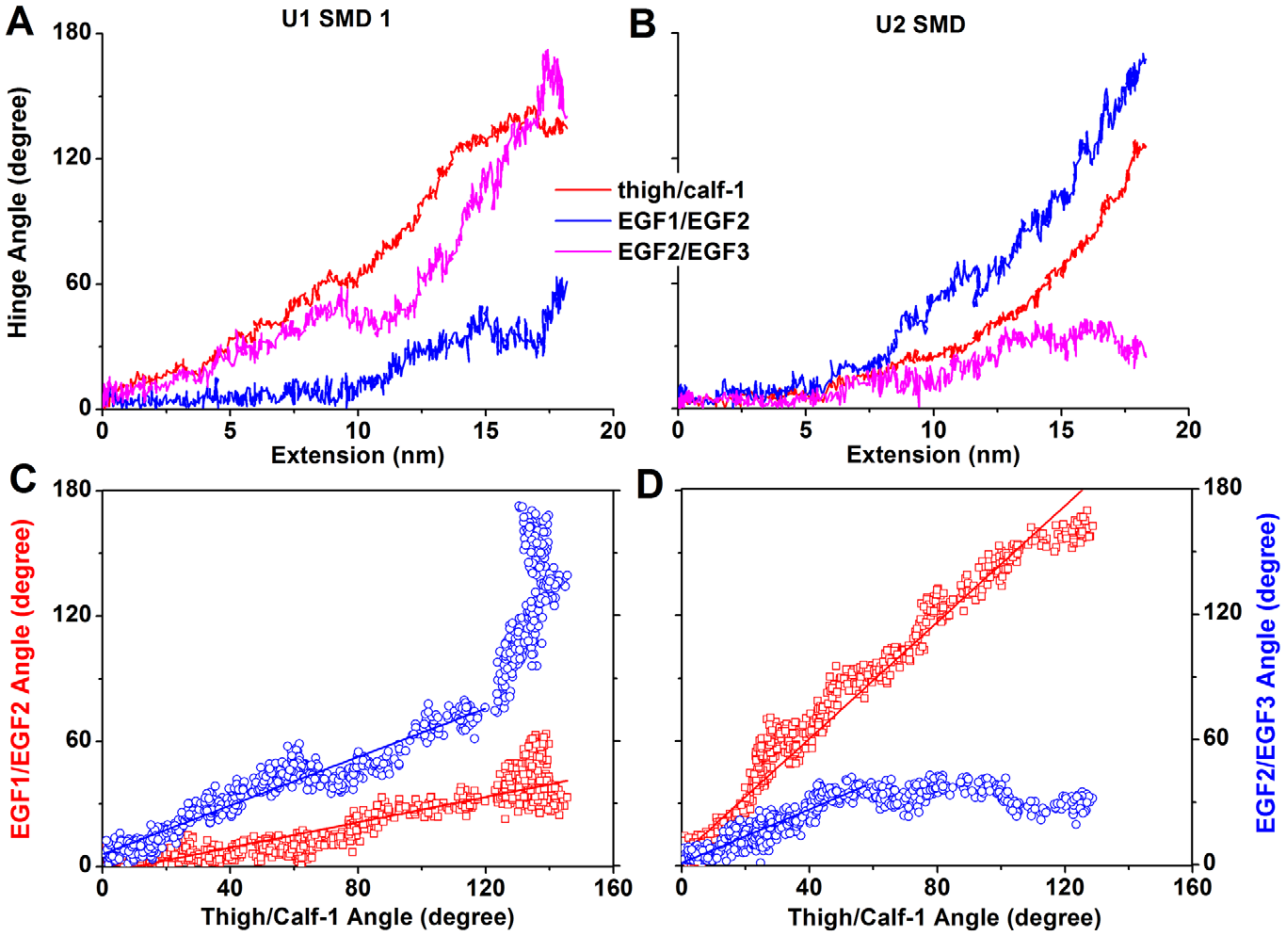
Changes in hinge angles at the α/β knees during unbending. **A & B**. Time courses of thigh/calf-1 (red), EGF1/EGF2 (blue), and EGF2/EGF3 (pink) hinge angles in the U1 SMD 1 (**A**) and U2 SMD (**B**). **C & D**. The EGF1/EGF2 (red squares) and EGF2/EGF3 (blue circles) hinge angles are plotted against the thigh/calf-1 hinge angle for the U1 SMD 1 (**C**) and U2 SMD (**D**). Solid lines are fits to the linear regions.

To examine whether the forced extension of the two knees occurred cooperatively, we analyzed three hinge angles: the thigh/calf-1 hinge angle for the α-knee and the EGF1/EGF2 and EGF2/EGF3 hinge angles for the β-knee (Figs. 5 and S3). Both the EGF1/EGF2 and EGF2/EGF3 hinges are bent in the starting structure of U1 (where the EGF1 and EGF2 domains were modeled) (Fig. S4); hence, these hinge angles opened significantly during integrin extension. In the starting structure of U2, by comparison, the β-knee is mainly bent at the EGF1/EGF2 hinge and hence this was where the major angle changes occurred. The hinge angles for the α- and β-knees increased with increasing head-tail extension concurrently (Figs. 5 and S3 A & B). To examine their cooperation, different hinge angles were plotted against each other, which reveal linear relationships until the integrin was over-stretched beyond the point where the integrin was fully straightened and the flexible EGF domains began to rotate about different hinges (Figs. 5 and S3 C & D). These results indicate that the interactions that hold the α/β knees in the bent conformation are cooperative.

### Unbending of unliganded integrin α_V_β_3_ by constant-force SMD

**Figure 6:**
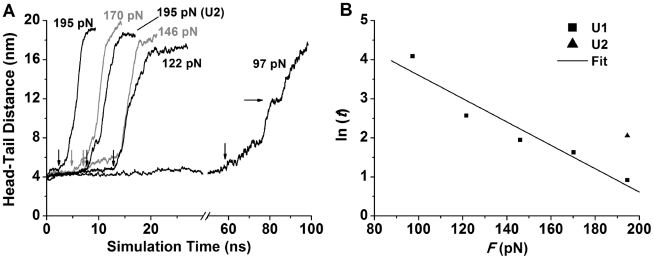
Constant-force SMD simulations. **A**. Head-tail distances vs. time of constant-force SMD simulations with the indicated forces. One simulation for U2 is indicated in parentheses and the others are for U1. Downward arrows indicate the moments when unbending started. A rightward arrow indicates an intermediate state. **B**. Natural log of waiting time *t* (in ns) for unbending vs. force data (symbols) and the Bell model fit (line) to the U1 data.

To complement and compare with the constant-velocity SMD simulations, we performed a series of constant-force SMD simulations for U1 and U2, where constant forces were loaded to the βA domain along the same direction with the βTD constrained as before. Instead of repeating the simulations at the same force, we used five forces from 97–195 pN for U1 and a 195 pN force for U2. Upon application of force, the head-tail distance was held at 4–5 nm initially, indicating the stability of the bent conformation, then increased suddenly, indicating a rapid conformational transition, and finally leveled off, indicating full extension (Fig. 6A). The rapid transition occurred after disrupting the interactions that gave rise to the major force peaks in the constant-velocity SMD simulations, confirming that these interactions provided the major barrier to unbending. In most cases, the interactions at both the hybrid/βTD and the hybrid/EGF4 interfaces were disrupted simultaneously. In the 146 and 97 pN constant-force simulations, however, the interactions at the hybrid/βTD interface were disrupted first, resulting in an initial slow-increase phase in the head-tail distance until the interactions at the hybrid/EGF4 interface were also broken. In the 122 pN constant-force simulation, the unbending process was slowed down in the middle of the conformational transition because of the interactions at the knees between the EGF1 and EGF2 domains and between the calf-1 and calf-2 domains. Interestingly, during the simulation with the lowest force of 97 pN, an intermediate state was observed, manifesting as a short plateau with a head-tail distance of ∼12 nm (Fig. 6A, rightward arrow). In the intermediate state the interactions at both the hybrid/βTD and hybrid/EGF4 interfaces were disrupted, but the interactions near the knees were still held.

Modeling integrin unbending as a rapid transition between two stable states separated by a barrier, the transition rate constant should be inversely proportional to the time required to overcome the major barrier. For the five U1 simulations, it is evident that this waiting time is shortened by increasing force (Fig. 6A, downward arrows), demonstrating that force accelerates unbending. This is consistent with the Bell model, which assumes that force lowers the barrier to exponentiate the transition rate [26]. Indeed, a plot of the natural log of the waiting time (ln*t*) vs. pulling force (*F*) appeared as a straight line (Fig. 6B) well-fitted by the Bell equation [26], *t* = *t*_0_exp(−*F*Δ*x*/*k*_B_ *T*), where *t*_0_ = 728±539 ns is the waiting time at zero force and Δ*x* = 1.2±0.2 Å is the width of the energy well that kinetically traps the integrin in the bent conformation. A similar head-tail distance vs. simulation time curve was obtained for the U2 simulation at 195 pN, although a longer waiting time was required to overcome the major barrier than the U1 simulation with the same force (Fig. 6). Taking at the face value, the *t*_0_ value suggests that integrin α_V_β_3_ could spontaneously overcome the major barrier to unbending on a time scale of 1 µs simply by thermally-excited Brownian motions.

### Stabilities of different conformations along the unbending pathway

**Figure 7:**
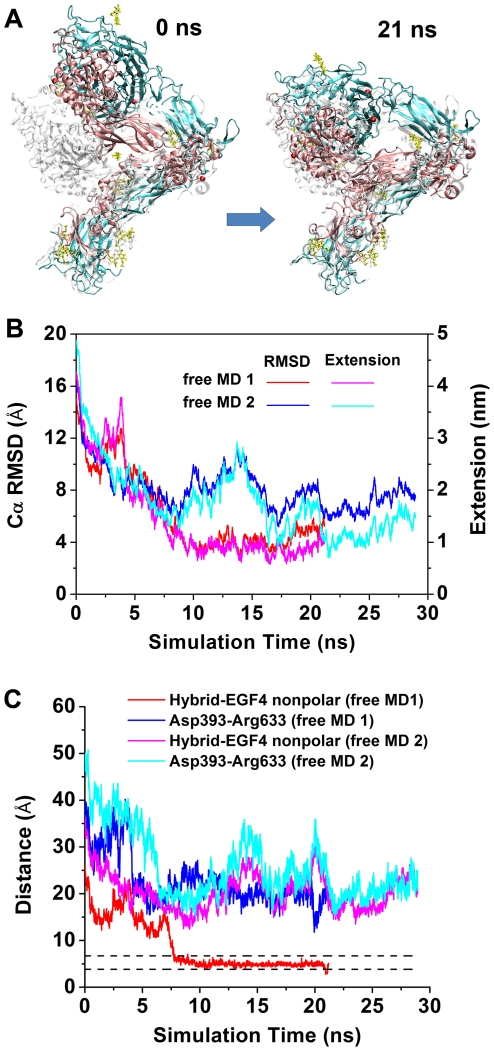
Free MD simulations of a partially-extended conformation. **A**. Snapshots of the U1 free MD 1 at indicated times showing rebending from a partially-extended integrin (colored) to the equilibrated bent conformation (gray). **B**. Time courses of Cα RMSDs relative to the equilibrated bent structure (red and blue, left ordinate) and head-tail extensions (magenta and cyan, right ordinate) in the U1 free MD 1 (red and magenta) and 2 (blue and cyan). **C**. Distances between the interacting residues, the nonpolar group (red and magenta) and the Asp393-Arg633 salt bridge (blue and cyan) at the respective hybrid/βTD and hybrid/EGF4 interfaces vs. simulation time of the U1 free MD 1 (red and blue) and 2 (magenta and cyan). As references, the upper and lower dashed lines indicate the respective distances of the nonpolar group and the Asp393-Arg633 salt bridge for the equilibrated bent U1 structure.

We performed two sets of free dynamics to examine the stabilities of integrin conformations along the unbending pathway after overcoming the major barrier. The first set studied a partially-extended conformation obtained right after the major force peak. Two structures from the respective trajectories of the U1 SMD 1 and U2 SMD 2 at ∼5 nm extensions were selected as starting structures for free MD 1 & 2, respectively (Fig. 2F). In the free MD simulations, the force at the βA domain was turned off. The constraint at the βTD was released in the free MD 1 but maintained in the free MD 2. In both simulations, the integrin gradually bent back (Fig. 7A and Video S3) as indicated by the decreased Cα RMSD relative to the equilibrated bent conformation and the reduced head-tail extension (Fig. 7B). This is particularly evident in the free MD 1 where the Cα RMSD dropped to ∼4 Å within 21 ns and the integrin returned nearly completely to its bent conformation. Not only did the overall structure bend back, but some important interactions between the headpiece and tailpiece that were broken during the simulated unbending were also recovered. Two groups of interactions, including the polar interactions between the hybrid domain and βTD and the nonpolar interactions between the hybrid and EGF4 domains, were gauged by the respective distances between the center of mass (COM) of the two sidechain oxygen atoms of Asp393 and the COM of the three sidechain nitrogen atoms of Arg633 and between the COM of the sidechains of Leu375, Ile380, and Leu383 and the COM of the sidechains of Met568, Leu573, and Leu574. These two distances were reduced when the integrin bent back (Fig. 7C). In free MD 1, the distance of the nonpolar interactions suddenly dropped to ∼4 Å and persisted to the end of the simulation, indicating that the nonpolar interactions between the hybrid and EGF4 domains were recovered. These results demonstrated again the importance of the nonpolar interactions in stabilizing the bent conformation. By comparison, the polar interactions were not recovered in the simulations because the Asp393-Arg633 distance was still much larger than that in the bent integrin (Fig. 7C).

**Figure 8:**
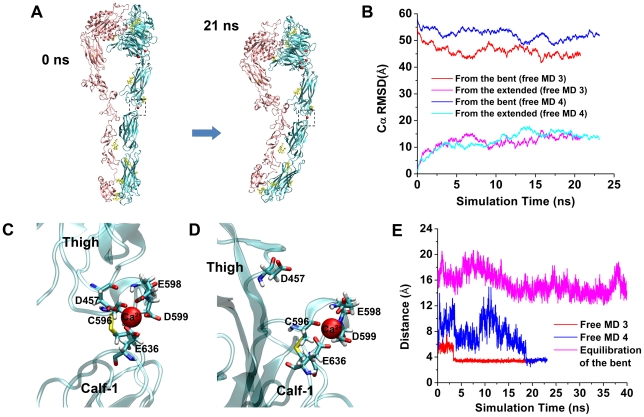
Free MD simulations of a fully-extended conformation. **A**. Snapshots of the U1 free MD 3 at indicated times showing the relaxation of a fully-extended integrin. Dashed boxes indicated the location of the α-knee Ca^2+^. **B**. Time courses of Cα RMSDs relative to the equilibrated bent structure (red and blue) or the starting fully-extended structure (magenta and cyan) in the U1 free MD 3 (red and magenta) and 4 (blue and cyan). **C** & **D**. Metal-ion coordination at the α-knee of the extended U1 at the end of the free MD 3 (**C**) or the bent U1 at the end of equilibration (**D**). Sticks represent residues and the red sphere represents the α-knee Ca^2+^. **E**. Time courses of distances between Asp457 of the α_V_ subunit and the α-knee Ca^2+^ in the U1 free MD 3 (red), the U1 free MD 4 (blue), and the equilibration of the bent U1 (magenta).

The second set of stability analyses was performed on a fully-extended conformation. Two simulations, free MD 3 & 4, were run with their starting structures selected from the trajectories of the U1 SMD 1 & 2, respectively, at ∼16 nm extensions (Fig. 2F). Free MD began after turning off the pulling force on the βA domain. The constraint at the βTD was released in the free MD 3 but maintained in the free MD 4. In both simulations, the integrin was relaxed and slightly bent back, but remained in a globally extended conformation for >20 ns (Fig. 8A and Video S4). Both Cα RMSDs measured from the equilibrated bent conformation and from the starting extended conformation reached plateaus after ∼5 ns (Fig. 8B). Interestingly, in both simulations, Asp457 of the thigh domain moved to coordinate with the Ca^2+^ ion at the genu of the α_V_ subunit (Fig. 8C), as observed by a sudden drop in the distance between the COM of the two Asp457 sidechain oxygens and this Ca^2+^ ion to <4 Å at ∼3 or ∼18 ns, respectively, which persisted throughout the remaining simulations (Fig. 8E), indicating the stability of the new coordination once it was formed. It should be noted that in the bent conformation, Asp457 was too far away (∼14 Å) to coordinate with the α_V_-genu Ca^2+^ (Fig. 8D). Only after integrin extension was it physically possible to interact with the α_V_-genu Ca^2+^. These results suggest a role for this newly-formed coordination involving Asp457 in stabilizing the extended conformation.

### Unbending of liganded integrin α_V_β_3_ by pulling its bound cyclic RGD ligand

**Figure 9:**
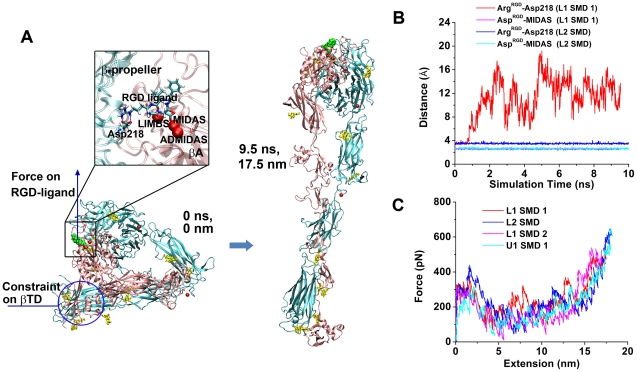
Forced unbending of liganded integrin α_V_β_3_. **A**. Snapshots of a liganded integrin α_V_β_3_ in the bent and fully-extended conformations at the indicated times and extensions in the L1 SMD 1. The βTD was constrained as shown by a circle. The RGD ligand (green spheres, same color coding and representations in all figures and videos) where force was loaded was zoomed-in to show the ligand binding site with a different orientation for better display, where the RGD ligand is shown as sticks and three Mg^2+^ ions as red spheres. LIMBS, ligand-associated metal binding site; ADMIDAS, adjacent to MIDAS. **B**. Time courses of distances between the ligand Arg and the α_V_ Asp218 (red and blue) or between the ligand Asp and the α_V_ MIDAS Mg^2+^ (magenta and cyan) in the L1 SMD 1 (red and magenta) and L2 SMD (blue and cyan). Some of the curves were obscured due to overlapping. **C**. Force-extension curves for the indicated SMD simulations. The U1 SMD 1 curve was replotted for comparison.

We next performed constant-velocity SMD simulations to unbend the liganded integrin α_V_β_3_ (L1 or L2) by pulling its bound cyclic RGD ligand away from the constrained βTD domain (Fig. 9A). In the L1 SMD 1 and L2 SMD, the integrin readily unbent with the ligand remained bound within 10 ns (Fig. 9A and Video S5 & S6). Compared to the unliganded integrin, the liganded integrin also reached a fully extended conformation with a closed headpiece, but the legs showed a greater degree of relative rotation.

In the initial structure, the RGD peptide interacted with integrin α_V_β_3_ via the ligand Asp sidechain oxygen coordinating with the metal ion in the βA domain metal ion-dependent adhesion site (MIDAS) and via the ligand Arg sidechain nitrogen forming a bifurcate H-bond with the Asp218 sidechain oxygen of the α_V_ β-propeller domain (Fig. 9A). The ligand Asp-MIDAS interaction remained intact throughout both simulations for L1 and L2, as indicated by the ∼3 Å stable distance between the COM of the two ligand Asp sidechain oxygens and the MIDAS (Fig. 9B). By comparison, the bifurcate ligand Arg-Asp218 H-bond was intact in the L2 SMD simulation (<4 Å stable distance between the COM of the three ligand Arg sidechain nitrogens and the COM of the two Asp218 sidechain oxygens throughout the simulation) but was disrupted in the L1 SMD 1 simulation. Nevertheless, the successful unbending of integrin α_V_β_3_ by force applied via a bound ligand suggests that the ligand-integrin α_V_β_3_ interaction is stronger than the interaction between the integrin headpiece and tailpiece.

The force-extension curves of the SMD simulations of L1 and L2 displayed a major force peak similar to that observed in the SMD simulations of U1 and U2 (Fig. 9C). Additionally, we observed two smaller peaks in the L1 SMD 1 and one smaller peak in the L2 SMD. Inspection of the changes of buried SASAs between the headpiece and tailpiece of integrin α_V_β_3_ revealed that the second peak in the L1 SMD 1 was due to interactions between the hybrid and EGF3 domains while the third peak in the L1 SMD 1 and the second peak in the L2 SMD were both due to interactions at the α/β knees (Fig. S5). Finally, we performed one more constant-velocity SMD simulation on L1 (L1 SMD 2) by pulling the same βA domain as in the SMD simulations of U1 and U2 (instead of pulling on the RGD ligand as in the L1 SMD 1 and L2 SMD). This time only the first peak was seen on the force-extension curve (Fig. 9C). These results suggest that the force direction plays a role in the determination of the unbending pathway after the first major force peak.

To examine the stability of the liganded integrin after extension, we performed two free MD simulations for the final structures of the L1 SMD 1 and L2 SMD but did not observe the formation of the α_V_ Asp457-Mg^2+^ coordination at the α-knee (Ca^2+^ in the unliganded integrin was replaced by Mg^2+^ in the liganded integrin). This may be due to the different post-equilibration structures of the unliganded and liganded integrins. Despite that the βA domain was pulled the same way in the L1 SMD 2 as the previous simulations of the unliganded integrin, the α_V_ Asp457 was far away from the α-knee Mg^2+^ in the resulting extended liganded integrin. By comparison, the α_V_ Asp457 was very close to the α-knee Ca^2+^ in the unliganded integrin after extension even before further equilibration. We note that without the α_V_ Asp457/α-knee Mg^2+^ coordination, the α-knee tended to rebend because the thigh/calf-1 hinge angle decreased significantly in one of the two free dynamics simulations of the liganded integrin (L1 free MD) (Fig. S6). With the α_V_ Asp457/α-knee Ca^2+^ coordination, by comparison, the thigh/calf-1 hinge angle remained stable at certain levels in the two free dynamics simulations of the extended unliganded integrin (U1 free MD 3 & 4). The differential stabilities of the α-knee in the absence and presence of the α_V_ Asp457/α-knee metal ion coordination supports the assertion that this metal ion coordination stabilizes, at least partly, the extended conformation.

## Discussion

X-ray crystallographic [4]–[10], electron microscopic [11]–[13], and antibody epitope mapping [27] studies have shown different static conformations of integrins corresponding to their different affinity states. The MD simulations reported here have connected the static conformations with dynamic processes, added time and force information, and provided structural insights to the continuous conformational changes of the integrin α_V_β_3_ ectodomain. Our results suggest that tensile forces applied to either the βA and/or β-propeller domains (Fig. 2) or the bound cyclic RGD ligand (Fig. 9) can easily induce the change of integrin α_V_β_3_ from a bent to an extended conformation. This work represents the first study in which the dynamic process of unbending of a complete integrin ectodomain was simulated in atomic details. Because no major distortions of individual domains resulted from pulling, our simulated structures of extended integrins should be more accurate than models obtained by simply rotating the bent crystal structures at the knees [8] or fitting various domains from the bent crystal structures into low resolution EM images of extended integrins [28]. Furthermore, that force can easily extend an integrin suggests an intriguing possible mechanism of force-induced integrin activation. Indeed, shear stress applied to lymphocytes promotes robust integrin-mediated adhesion [16]. In addition, either intracellular force by the cytoskeleton or extracellular force by shear strengthens adhesions mediated by integrin α_5_β_1_-fibronection bonds [17]. Moreover, recent single-bond experiments have demonstrated catch bond behavior (such that force prolongs bond lifetime) for bonds between integrin α_5_β_1_ and fibronectin [18] and between integrin α_L_β_2_ and intercellular adhesion molecule 1 [19].

Although tensile forces readily unbent integrin α_V_β_3_, we did not observe hybrid domain swing-out, a key conformational change in models of integrin activation [10], [11], in the SMD simulations (Figs. 2 and 9) or free dynamics simulations of the extended integrin with closed legs (Fig. 8). This result differs from previous SMD simulations on the α_V_β_3_ headpiece [20], [21], where pulling the ligand binding site at the βA domain and the hybrid domain C-terminus induced the hybrid domain to swing out. Intuitively, when tensile forces apply to the V-shape α_V_β_3_ headpiece, the hybrid domain would naturally swing out to open the headpiece. Pulling the whole ectodomain would be quite different. In the SMD simulations of an extended model of α_IIb_β_3_, a lateral force opened the closed α/β legs and induced the hybrid domain to swing out, but an axial force pulling along the straight integrin kept the hybrid domain closed [8]. In our SMD simulations with only axial forces, the legs remained closed and the hybrid domain did not swing out. It is not clear whether axial and lateral forces would apply to integrins simultaneously or sequentially under physiological situations. However, we observed extensive contacts between the two α/β legs that were stronger than those between the headpiece and tailpiece (data not shown), suggesting that opening the closed legs may cost higher energy than unbending. Therefore, different forces may be required to first unbend an integrin and then open the legs and the hybrid domain. There may be mechanisms other than unbending and hybrid domain swing-out for force to increase binding affinity and/or bond lifetime of integrins with ligands. Indeed, lifetimes of α_5_β_1_-fibronectin [18] and α_L_β_2_-intercellular adhesion molecule 1 [19] bonds have been observed to be prolonged by axial instead of lateral forces regardless of whether the integrins were unbent (and/or the hybrid domain was swung out) by force or the integrins had already been extended (and/or the hybrid domain had already been swung out) prior to the application of force.

We identified two groups of critical interactions between the headpiece and tailpiece of the β_3_ subunit (Fig. 4) that form the major energy barrier to the unbending of integrin α_V_β_3_. One group mainly consists of polar interactions between the hybrid domain and the βTD, including several H-bonds and one salt bridge between Asp393 and Arg633. Indeed, Arg633 has been identified as a key residue to keep integrin α_V_β_3_ in the bent conformation by NMA [24]. To verify the importance of Arg633, the authors further showed experimentally that its deletion or substitution by Ala enhanced α_IIb_β_3_-fibrinogen binding. The other group of critical interactions includes hydrophobic interactions between the hybrid and EGF4 domains. Not only did these interactions contribute to the major force peak resisting unbending, but they were also seen to reform during rebending of the partially-extended integrin (Fig. 7). These results predict that disrupting the hydrophobic interactions by mutating the residues involved, including Leu375, Ile380, Leu383, Met387, Met568, Leu573, and Leu574, to polar residues will promote extension of β_3_ integrins. Besides the two groups of interactions identified from our simulations, there may be other interactions that play roles in the conformational changes of integrins. Indeed, interactions between a β hairpin protruding from the β-propeller domain and a region in the middle of the EGF3 and EGF4 domains have been shown as a clasp in restraining integrin activation [29]. This is consistent with our simulations showing that these interactions were maintained until the integrin was unbent.

Our constant-force SMD simulations indicate that the time required to overcome the major barrier to unbending follows the Bell model [26] (Fig. 6). The value of Δ*x* = 1.2±0.2 Å suggests a narrow energy well not very sensitive to force. The extrapolated zero-force waiting time of *t*_0_ = 728±539 ns suggests that the major barrier could be spontaneously overcome very rapidly on a time scale of 1 µs, allowing the integrin to reach a partially-extended conformation. It should be noted that these results are based on one simulation per force only, so the inferred zero-force waiting time may not be accurate. We also cannot exclude the possibility of a different unbending time-force relationship at lower forces. On the other hand, our free MD simulations suggested that the partially-extended integrin could bend back on a much shorter time scale of 10 ns (Fig. 7), so the rebending rate may be much faster than the unbending rate. Therefore, although unbending may occur spontaneously, the integrin may still far more likely assume the bent conformation at zero force. It may also undergo fast transitions between the bent and partially-extended conformation like “breathing”. Springer and coworkers propose that during integrin conformation “breathing”, epitopes on the β-leg may expose for antibody binding [11], [14]. The binding of an antibody acts as a wedge to keep the interface between the headpiece and tailpiece open, which stabilizes the extended conformation and thus activates integrins. Indeed, EM studies observe that a small group of integrin α_V_β_3_ under inactive conditions has a wider separation between the headpiece and tailpiece while the majority exhibits headpiece-tailpiece contacts [11]. Upon stimulations, the conformational equilibrium in a population of integrins may readily shift so that the extended conformation becomes more popular.

Another interesting observation is the formation of a new coordination between the thigh domain Asp457 and the metal ion at the α-genu when the unliganded integrin became fully extended (Fig. 8), although this was not observed in the unbending simulations of the liganded integrin probably due to the different starting structure and insufficient simulation time. Because the new coordination was very stable once it was formed (Fig. 8E) and the α-knee tended to rebend when the new coordination was not formed (Fig. S6), the new coordination likely plays a role in stabilizing the extended conformation. In addition, both Asp457 and the α-genu metal ion site are conserved across species (Fig. S7A), suggesting a functional role. While future mutagenesis experiments are required to provide definitive tests, the putative coordination between Asp457 and the α-genu metal ion upon integrin extension may explain why the majority of α_V_β_3_ integrins adopt bent conformations in Ca^2+^ but extended conformations in Mn^2+^ [11]. The explanation assumes different propensities for distinct metal ions to coordinate with Asp457 such that distinct metal ion conditions favor different fractions of bent vs. extended integrins. It should be noted that among all 18 known human α subunits, residue 457 has wide variations, although the α-genu metal ion site is largely conserved (Fig. S7B). Only in several α subunits, including α_5_, α_7_, and α_8_, is Asp457 conservatively substituted by Glu. Therefore, the putative Asp457/α-genu metal ion coordination is likely a specific property for a subset of integrins.

It remains debatable whether the deadbolt or switchblade model describes the mechanism of integrin activation [2]. Our simulations may seem to favor the switchblade model because force induces a switchblade-like extension. However, initial ligand binding before force loading could be regulated by a “deadbolt”. Future experimental and computational studies are needed to clarify the mechanism of integrin activation.

## Methods

### System setup

Four systems were set up for MD simulations: U1, U2, L1, and L2 (Table S1). U1 was modeled after the early crystal structure of the unliganded integrin α_V_β_3_ ectodomain (PDB code 1U8C) [6] available at the time when we started this study, in which the EGF1 and EGF2 domains at the genu of the β_3_ subunit were unresolved. To complete the ectodomain, we used MODELLER [30] to build a homology model for the two missing domains based on the crystal structure of a fragment of the β_2_ subunit (PDB code 2P28) [31] and used TMD to fit it to the crystal structure (Text S1 and Fig. S8). The modeled EGF1 and EGF2 domains plus the PSI domain from the 1U8C structure were then added to the liganded integrin α_V_β_3_ ectodomain (PDB code 1L5G) [5] to obtain L1. U2 is the recently released crystal structure of the unliganded integrin α_V_β_3_ ectodomain plus a short α/β transmembrane fragment, where the EGF1 and EGF2 domains are visible (PDB code 3IJE) [7]. Wherever resolvable, the 1U8C structure is very close to the 3IJE structure. Our homology model of the EGF1 and EGF2 domains are similar to those of the 3IJE structure in orientation and majority of the tertiary structures, with noticeable variations only in the loops and the linker between the two domains (Fig. S4). L2 was obtained by extracting the EGF1, EGF2, and PSI domains from the 3IJE structure and adding them to the 1L5G structure. Therefore, U1 and U2 are highly comparable except for the EGF1 and EGF2 domains; so are L1 and L2. For L1 and L2, we replaced nonstandard N-Methylvaline and D-form Phenylalanine in the cyclic-RGD ligand to standard Valine and L-form Phenylalanine, respectively. We also replaced Mn^2+^ ions with Mg^2+^ because force field parameters for Mn^2+^ were not available.

We employed LEaP in AMBER8 package [32] to prepare solvated structures with TIP3P [33] water boxes (Fig. 1B). The closest distance between the walls of the water boxes and the proteins was 15 Å while the closest distance between water molecules and proteins was set to 1 Å. Four water boxes with different dimensions were set up for the four structures (Table S1). Na^+^ and Cl^−^ ions were added to neutralize the systems at a 150 mM salt concentration. After equilibration, the water boxes were further enlarged for SMD simulations (Fig. 2A) and more Na^+^ and Cl^−^ ions were added accordingly to maintain the 150 mM salt concentration.

### MD simulations

MD simulations were performed using NAMD [34]. Duan et al. [35] and GLYCAM04/06 [36], [37] force fields were used for amino acids and carbohydrates attached to the proteins, respectively. We used 2 fs time step, 12 Å cutoff for non-bonded force, and 10 Å switching distance for smoothing functions of non-bonded force. Bonds involving hydrogen were set rigid by using the SHAKE algorithm [38] for protein and the SETTLE algorithm [39] for water. Periodic boundary conditions were applied and the Particle Mesh Ewald method [40] was used to calculate full electrostatic interactions every 4 fs. Atomic coordinates of the systems were saved every 1 ps.

First, each system was energy-minimized for three consecutive 10,000 conjugate-gradient steps: first with all protein atoms fixed, second with only the backbone atoms fixed, and third with all atoms free. Then, the systems were gradually heated from 0 to 300 K in 120 ps with constraints of 1 kcal mol^−1^ Å^−2^ spring constants applied on all protein atoms under constant volume. Next, pressure was adjusted to 1 atm in 100 ps with the Langevin piston method [41] under constant temperature controlled with Langevin dynamics. After that, the constraints on the protein atoms were gradually released in 100 ps under constant volume and constant temperature (NVT). Finally, 40 (or 50) ns equilibrations were performed for U1 and L1 (or U2 and L2) under constant pressure and constant temperature (NPT). The pressure was maintained at 1 atm by the Langevin piston method while the temperature was maintained at 300 K by Langevin dynamics with a damping coefficient of 5 ps^−1^.

After equilibration in the small water boxes (cf. Fig. 1B), more water molecules were added to enlarge the water boxes (cf. Fig. 2A). For equilibration of the enlarged water boxes, the systems were first energy-minimized for 10,000 conjugate-gradient steps with all protein atoms fixed, next heated from 0 to 300 K in 120 ps with spring constraints of 1 kcal mol^−1^ Å^−2^ spring constant on all protein atoms under constant volume followed by 2–4 ns equilibration in NPT ensembles, then set free by gradually releasing constraints on the protein atoms in 100–400 ps followed by 2–4 ns final equilibration in NPT ensembles. The enlarged systems were then ready for SMD simulations. In all production SMD and free MD simulations, the controls on pressure and temperature were turned off so as to reduce disturbance on the dynamics.

In SMD simulations, a group of Cα atoms were selected. Then force or constraint was exerted on the COM of the selected atoms. When different domains were pulled or constrained, the residues were selected as follows: βA residues 113–117, 151–156, 190–197, 244–250, 306–310, and 329–332; β-propeller residues 22–26, 97–101, 160–164, 225–229, 279–283, 343–347, and 407–411; βTD residues 610–620, 639–642, 656–658, 665–670, and 679–682; and all five residues of the cyclic RGD ligand. While force was on the pulling COM, a harmonic potential with a spring constant of 10 kcal mol^−1^ Å^−2^ was added to the constraint COM. In the constant-velocity SMD simulations [25], the pulling COM was harmonically constrained with a force, *F* = *k* (*vt*−*x*), where *k* is the spring constant, *v* is the pulling velocity, *t* is time, and *x* is the coordinate along the force direction. A total of ∼468 ns production simulations were performed, including 8 constant-velocity and 5 constant-force SMD simulations for U1, 1 constant-velocity and 1 constant-force SMD for U2, 2 constant-velocity SMD for L1, 1 constant-velocity SMD for L2, 4 free MD for U1, 1 free MD for L1, and 1 free MD for L2 (Table S1).

### Analysis of MD trajectories

VMD [42] was employed to analyze simulations, render molecular graphics, and generate trajectory videos. SASA was calculated with 1.4 Å probe radius. Buried SASA between two contacting portions was calculated as the difference between the SASAs of one portion without and with the other portion. Restricted to one domain during SASA calculation, the buried SASA of that domain was obtained. An H-bond was defined by a <3.5 Å donor-acceptor distance and a >120° donor-hydrogen-acceptor angle. Hinge angles were measured using Hingefind [43]. The various α_V_β_3_ domains are defined as follows: β-propeller, residues 1–438; thigh, residues 439–600; calf-1, residues 601–738; calf-2, residues 739–956; PSI, residues 1–57; hybrid, residues 58–110 and 354–434; βA, residues 111–353; EGF1, residues 435–472; EGF2, residues 473–522; EGF3, residues 523–559; EGF4, residues 560–599; β-ankle, residues 600–605; βTD, residues 606–690.

## Supporting Information

Figure S1 Changes in headpiece-tailpiece interactions near the major force peaks. Buried SASAs (upper row, left ordinate) and numbers of H-bonds (lower row, left ordinate) of hybrid (blue), βA (cyan), EGF4 (red), and βTD (magenta) domains as well as pulling force (gray, both rows, right ordinate) were plotted vs. extensions for the U1 SMD 2 (left column) and U1 SMD 3 (right column). Some of the curves were obscured due to overlapping.
(0.45 MB TIF)

Figure S2 H-bonds at the hybrid/EGF4 interface of U2. Stereoview of the post-equilibrated U2 structure at the interface between hybrid (orange) and EGF4 (tan) with residues involved in H-bonds (indicated by dashed lines) shown as sticks.
(1.74 MB TIF)

Figure S3 Changes in hinge angles at the α/β knees during unbending. A & B. Time courses of thigh/calf-1 (red), EGF1/EGF2 (blue), and EGF2/EGF3 (pink) hinge angles in the U1 SMD 2 (A) and 3 (B). C & D. The EGF1/EGF2 (red squares) and EGF2/EGF3 (blue circles) hinge angles are plotted against the thigh/calf-1 hinge angle for the U1 SMD 2 (C) and 3 (D). Solid lines are fits to the linear regions.
(0.50 MB TIF)

Figure S4 Comparison of the homology model of the EGF1 and EGF2 domains with the crystal structure. The homology model (red) and the crystal structure (blue, PDB code 3IJE) were aligned using Cα atoms of the β_3_ subunit other than the EGF1 and EGF2 domains (left and middle) or using the Cα atoms of the EGF1 and EGF2 domains (right).
(3.03 MB TIF)

Figure S5 Buried SASA between the headpiece and tailpiece during unbending of the liganded α_V_β_3_. Buried SASAs (colored, left ordinate) of the indicated domains were plotted vs. simulation time along with pulling force (gray, right ordinate) for the L1 SMD 1 and L2 SMD.
(0.47 MB TIF)

Figure S6 Changes of the thigh/calf-1 hinge angle in the free MD simulations of the extended integrin. The thigh/calf-1 hinge angles are plotted against simulation time for the indicated simulations.
(0.14 MB TIF)

Figure S7 Amino acid sequence alignment of the α_V_ subunit. The sequences were aligned across species (A) or human α family members (B) by using ClustalW2 [44]. The sequences were retrieved from UniProt Knowledgebase (UniProtKB) [45]. Conserved mutations at residue 457 and the α-genu metal ion site are highlighted. “*” indicates identical residues, “:” indicates conserved substitutions, and “.” indicates semi-conserved substitutions.
(6.59 MB TIF)

Figure S8 Building complete ectodomain models of integrin α_V_β_3_. A. Amino acid sequence alignment between β_2_ and β_3_ in the region containing the PSI, EGF1, and EGF2 domains with ClustalW2 [44]. B. Alignment of the template and the homology model. On the left, the hybrid and EGF3 domains (blue) from the crystal structure of integrin α_V_β_3_ (PDB code 1U8C) were aligned to the template (gray) of the β_2_ fragment (PDB code 2P28) before homology modeling. On the right, the final homology model (red) was compared to the template. C. Targeting the extended β_3_ model (red) to the bent β_3_ structure (gray) by TMD simulation. Snapshots were taken at indicated times. D. The final bent β_3_ model (red) in the bent α_V_β_3_ structure (α_V_, yellow; β_3_, gray). E. RMSD relative to the bent structure for all heavy atoms of the PSI, hybrid, and EGF3 domains during the TMD simulation. F. Comparison of the DOPE scores of the homology model and the template.
(5.24 MB TIF)

Table S1 Summary of MD simulations for integrin α_V_β_3_.
(0.04 MB DOC)

Text S1 Building complete ectodomain models of unliganded and liganded integrin α_V_β_3_.
(0.06 MB DOC)

Video S1 Unbending of unliganded integrin α_V_β_3_ in the U1 SMD 1. The βA domain was pulled vertically away from the constrained βTD. The running simulation time is indicated. The α_V_ subunit is in cyan and the β_3_ subunit is in pink. The same color codes are used in all videos.
(4.65 MB AVI)

Video S2 Unbending of unliganded integrin α_V_β_3_ in the U2 SMD. The βA domain was pulled vertically away from the constrained βTD.
(4.55 MB AVI)

Video S3 Rebending of partially-extended unliganded integrin α_V_β_3_ in the U1 free MD 1. The simulation started from the partially-extended structure that was selected from the U1 SMD 1. Nothing was constrained during the simulation. The bent structure is in gray for comparison.
(9.82 MB AVI)

Video S4 Relaxation of fully-extended unliganded integrin α_V_β_3_ in the U1 free MD 3. The simulation started from the fully-extended structure that was selected from the U1 SMD 1. Nothing was constrained during the simulation.
(5.82 MB AVI)

Video S5 Unbending of liganded integrin α_V_β_3_ in the L1 SMD 1. The cyclic RGD ligand (green spheres) was pulled vertically away from the constrained βTD.
(4.52 MB AVI)

Video S6 Unbending of liganded integrin α_V_β_3_ in the L2 SMD. The cyclic RGD ligand (green spheres) was pulled vertically away from the constrained βTD.
(4.72 MB AVI)
