# Supplementary figures and images for: Molecular Dynamics Simulations of Forced Unbending of Integrin αVβ3
Source: PLoS Comput Biol. 2011 Feb 17;7(2):e1001086. doi: 10.1371/journal.pcbi.1001086 (PMC3040657; doi:10.1371/journal.pcbi.1001086)

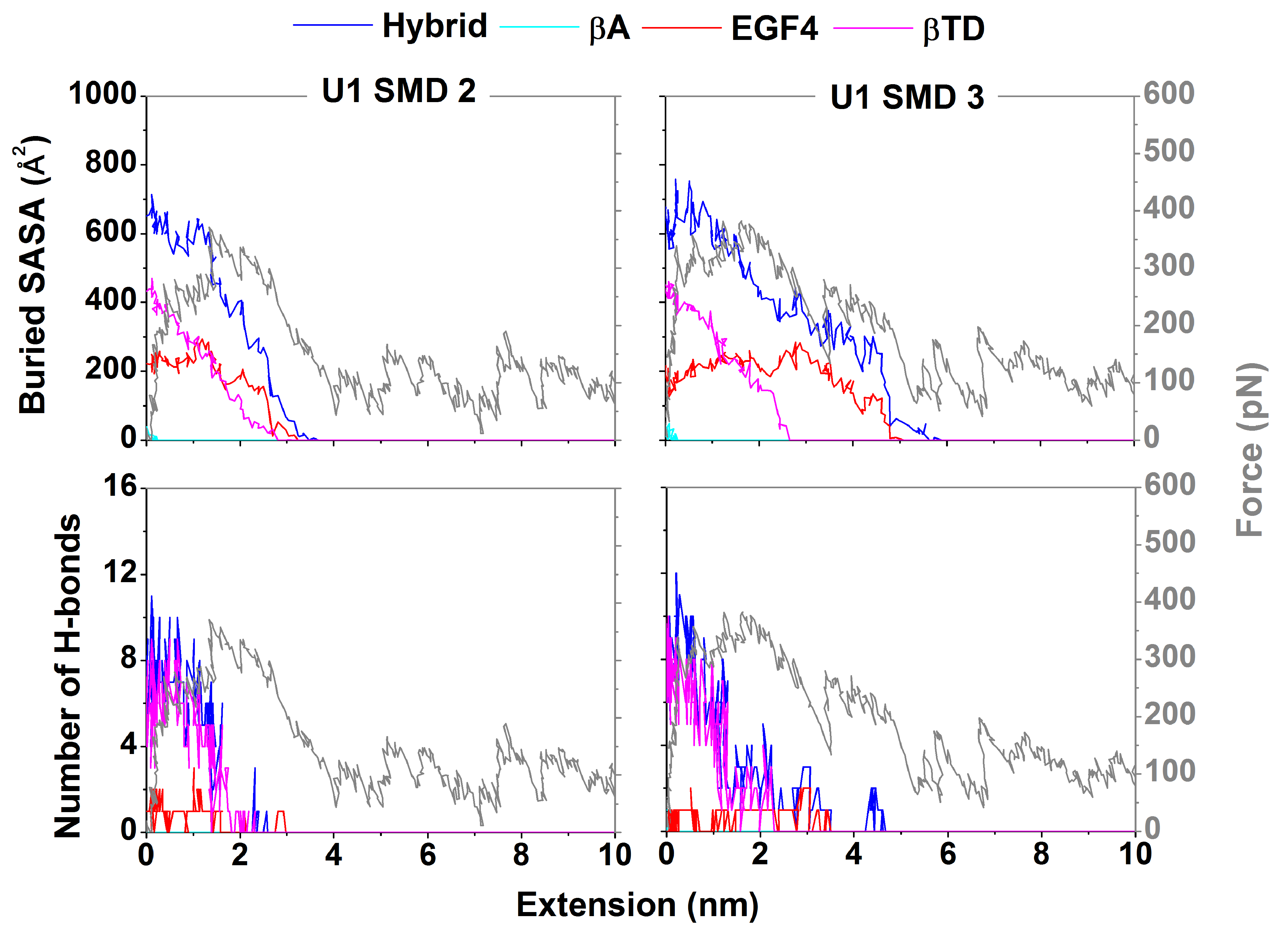

Supplement: Figure S1 — Changes in headpiece-tailpiece interactions near the major force peaks. Buried SASAs (upper row, left ordinate) and numbers of H-bonds (lower row, left ordinate) of hybrid (blue), βA (cyan), EGF4 (red), and βTD (magenta) domains as well as pulling force (gray, both rows, right ordinate) were plotted vs. extensions for the U1 SMD 2 (left column) and U1 SMD 3 (right column). Some of the curves were obscured due to overlapping. (0.45 MB TIF) [file pcbi.1001086.s001.tif]

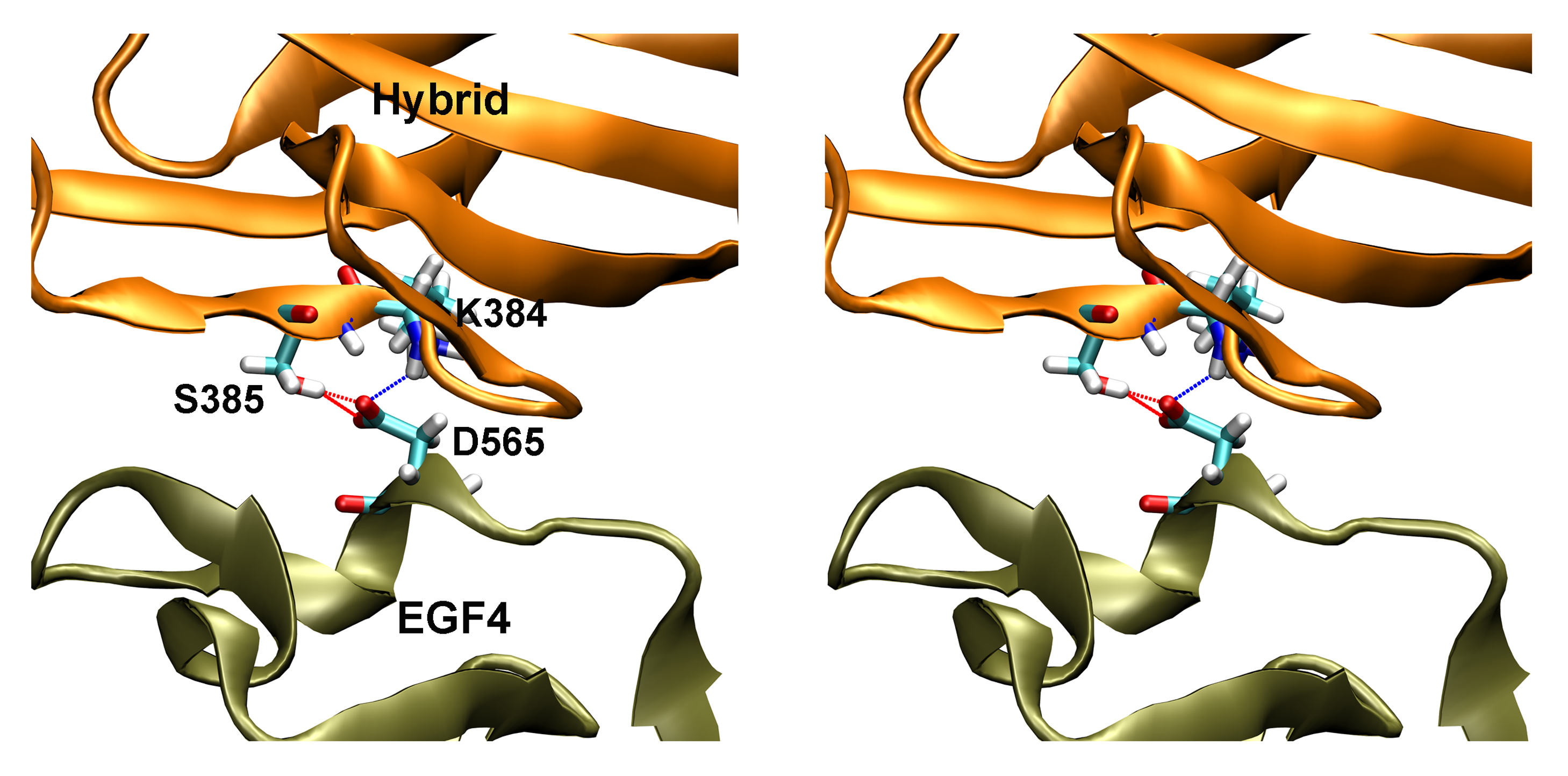

Supplement: Figure S2 — H-bonds at the hybrid/EGF4 interface of U2. Stereoview of the post-equilibrated U2 structure at the interface between hybrid (orange) and EGF4 (tan) with residues involved in H-bonds (indicated by dashed lines) shown as sticks. (1.74 MB TIF) [file pcbi.1001086.s002.tif]

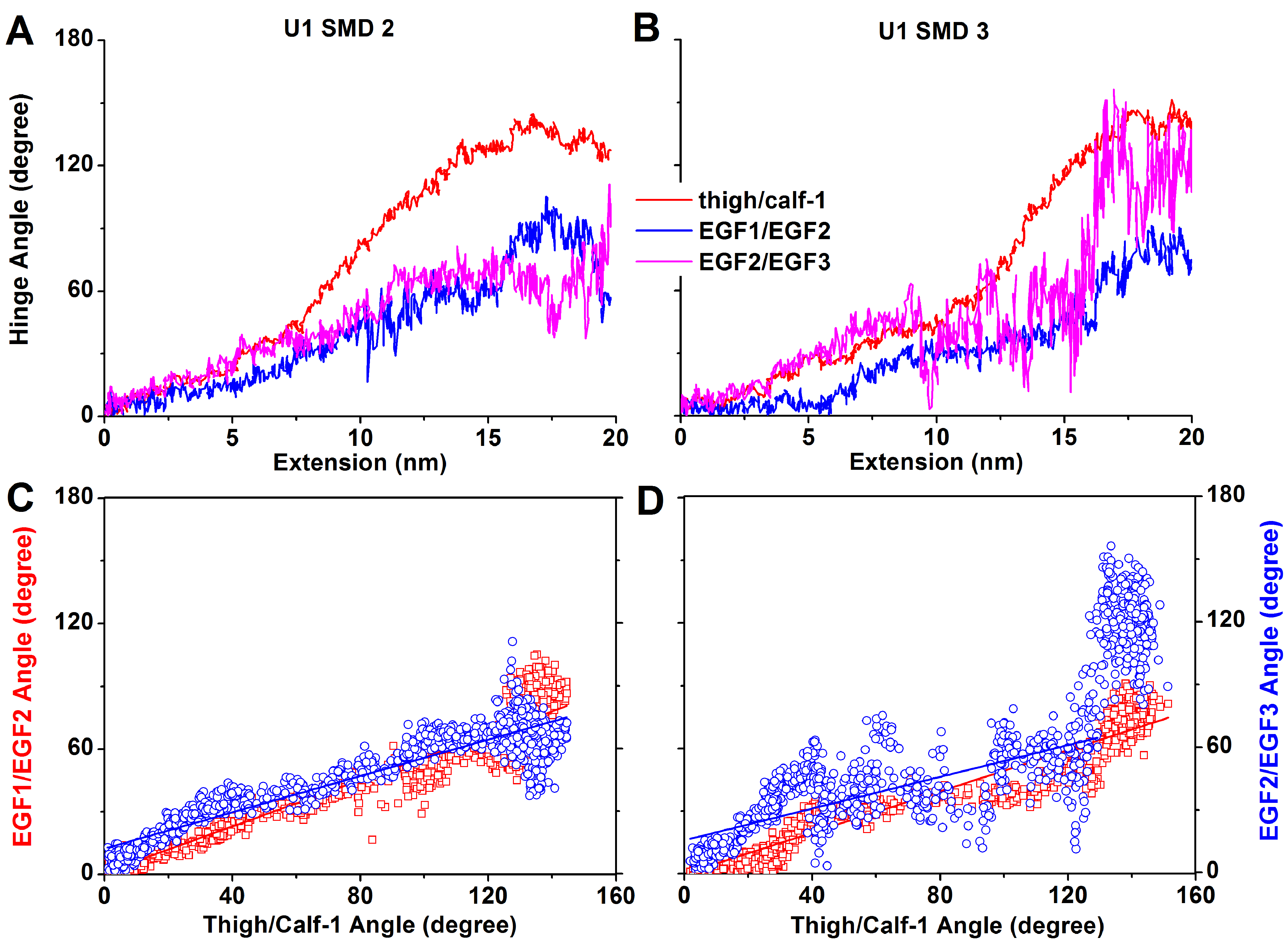

Supplement: Figure S3 — Changes in hinge angles at the α/β knees during unbending. A & B. Time courses of thigh/calf-1 (red), EGF1/EGF2 (blue), and EGF2/EGF3 (pink) hinge angles in the U1 SMD 2 (A) and 3 (B). C & D. The EGF1/EGF2 (red squares) and EGF2/EGF3 (blue circles) hinge angles are plotted against the thigh/calf-1 hinge angle for the U1 SMD 2 (C) and 3 (D). Solid lines are fits to the linear regions. (0.50 MB TIF) [file pcbi.1001086.s003.tif]

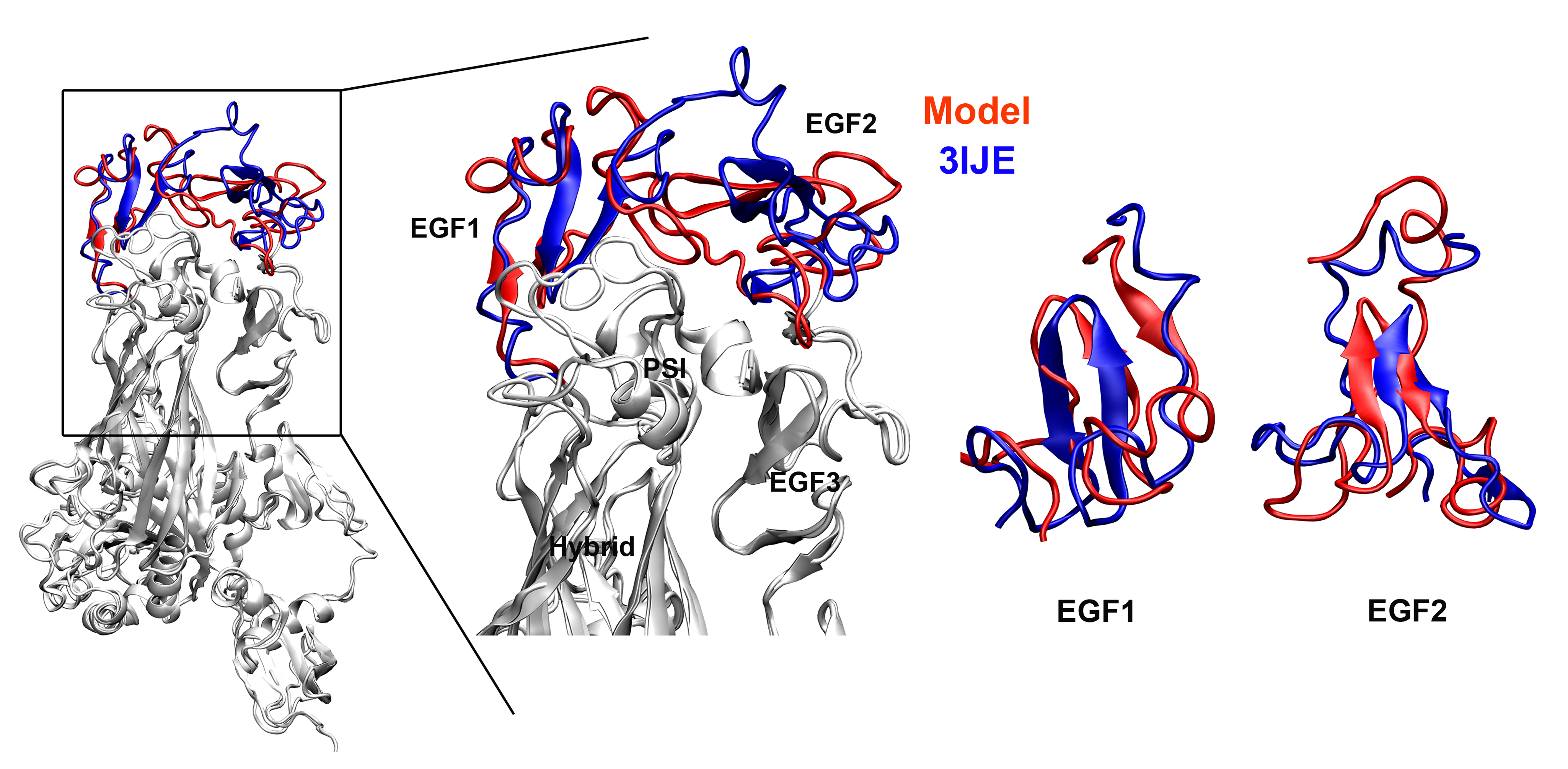

Supplement: Figure S4 — Comparison of the homology model of the EGF1 and EGF2 domains with the crystal structure. The homology model (red) and the crystal structure (blue, PDB code 3IJE) were aligned using Cα atoms of the β3 subunit other than the EGF1 and EGF2 domains (left and middle) or using the Cα atoms of the EGF1 and EGF2 domains (right). (3.03 MB TIF) [file pcbi.1001086.s004.tif]

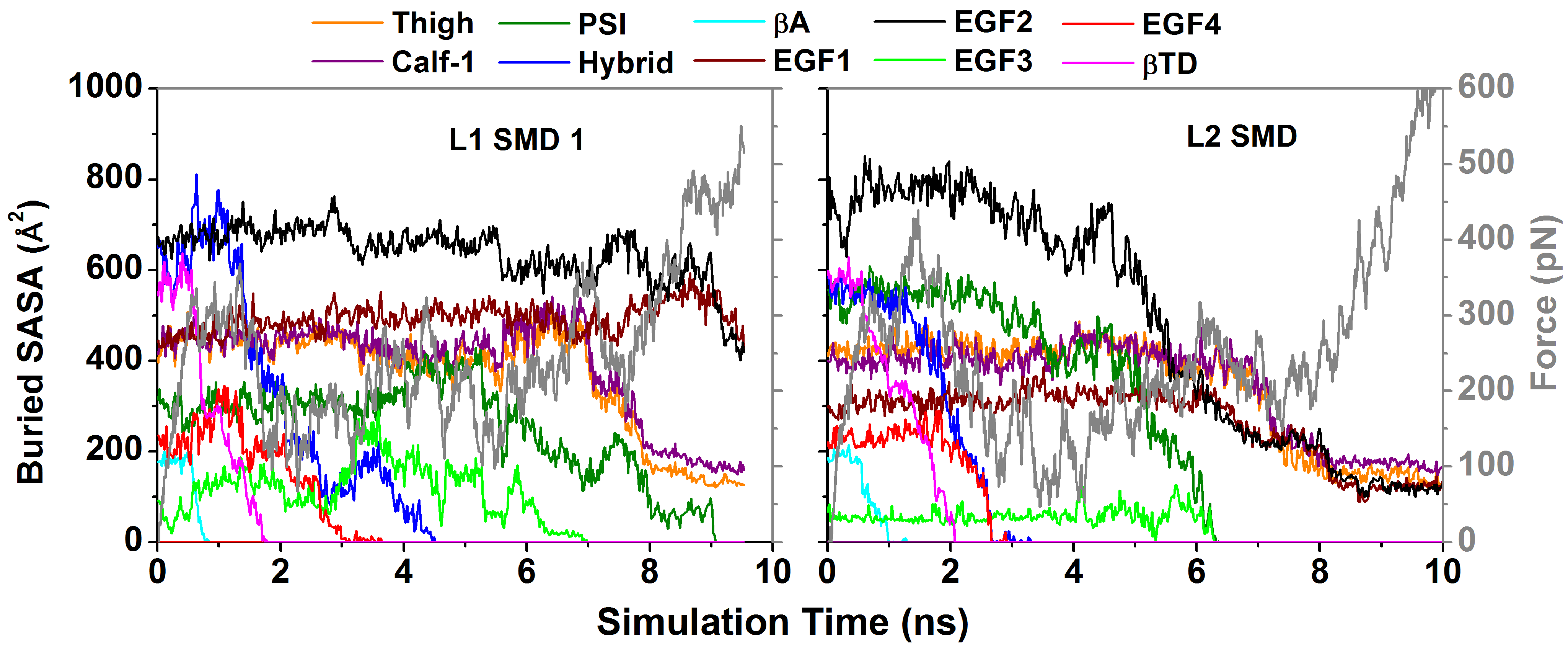

Supplement: Figure S5 — Buried SASA between the headpiece and tailpiece during unbending of the liganded αVβ3. Buried SASAs (colored, left ordinate) of the indicated domains were plotted vs. simulation time along with pulling force (gray, right ordinate) for the L1 SMD 1 and L2 SMD. (0.47 MB TIF) [file pcbi.1001086.s005.tif]

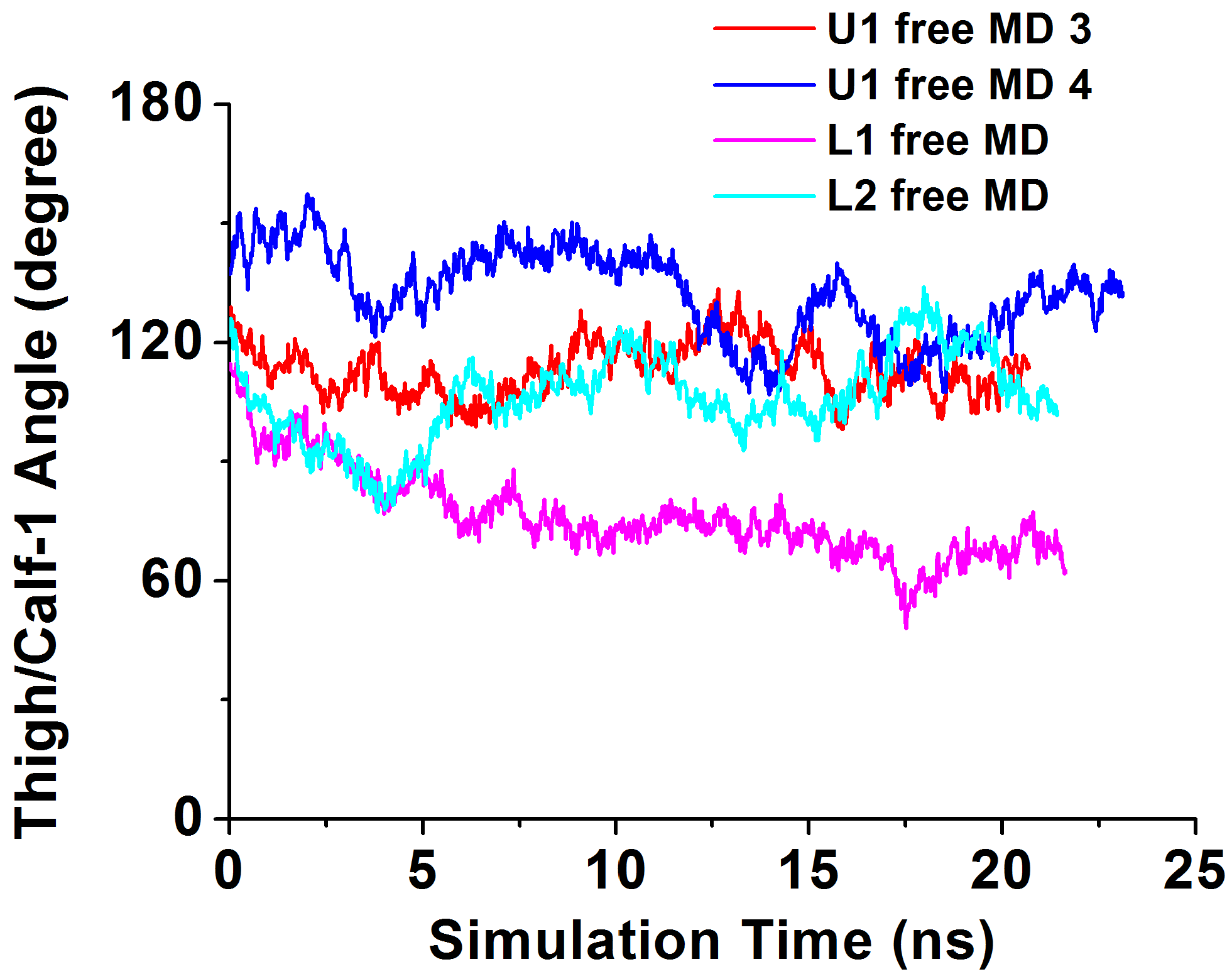

Supplement: Figure S6 — Changes of the thigh/calf-1 hinge angle in the free MD simulations of the extended integrin. The thigh/calf-1 hinge angles are plotted against simulation time for the indicated simulations. (0.14 MB TIF) [file pcbi.1001086.s006.tif]

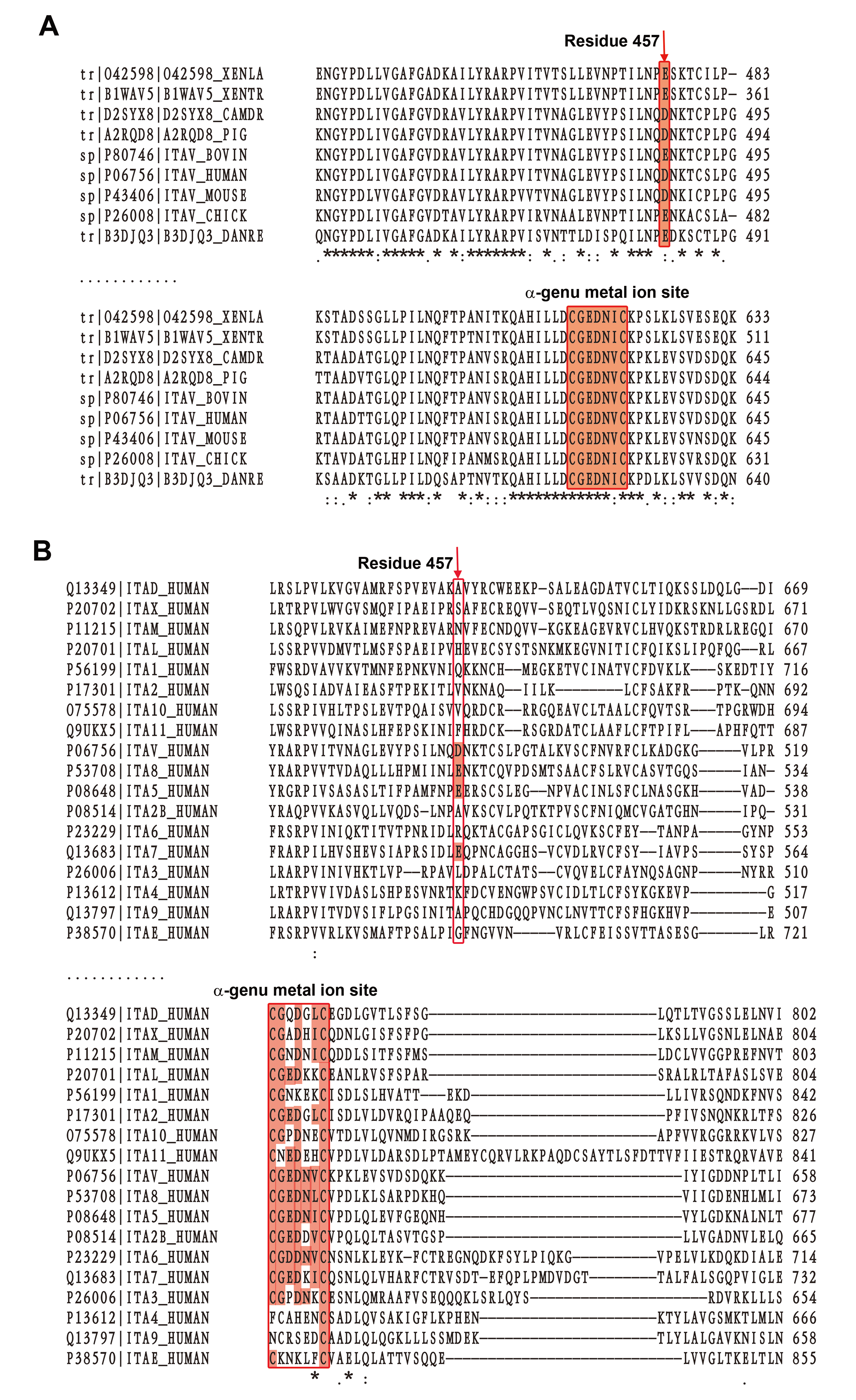

Supplement: Figure S7 — Amino acid sequence alignment of the αV subunit. The sequences were aligned across species (A) or human α family members (B) by using ClustalW2 [44]. The sequences were retrieved from UniProt Knowledgebase (UniProtKB) [45]. Conserved mutations at residue 457 and the α-genu metal ion site are highlighted. “*” indicates identical residues, “:” indicates conserved substitutions, and “.” indicates semi-conserved substitutions. (6.59 MB TIF) [file pcbi.1001086.s007.tif]

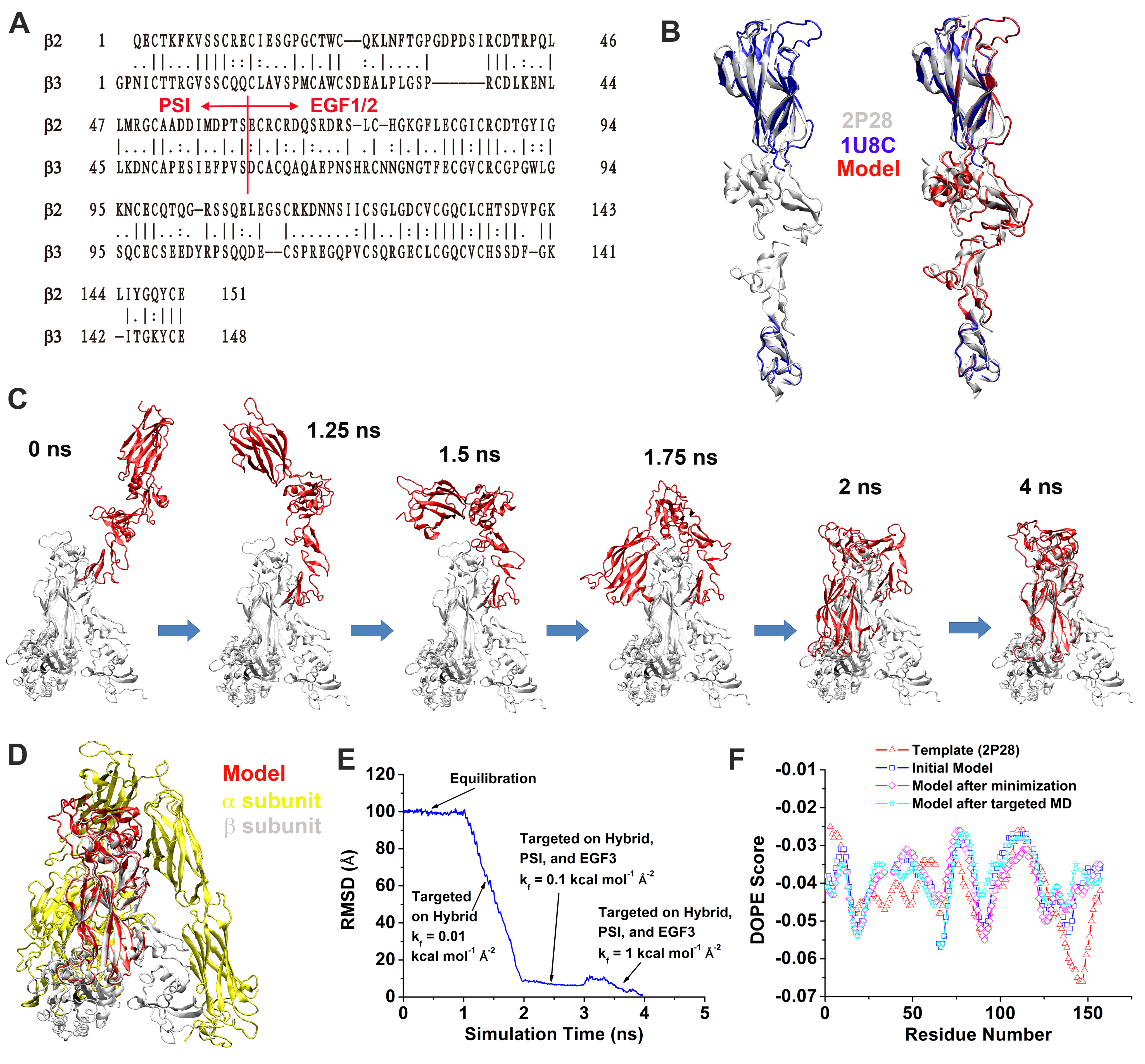

Supplement: Figure S8 — Building complete ectodomain models of integrin αVβ3. A. Amino acid sequence alignment between β2 and β3 in the region containing the PSI, EGF1, and EGF2 domains with ClustalW2 [44]. B. Alignment of the template and the homology model. On the left, the hybrid and EGF3 domains (blue) from the crystal structure of integrin αVβ3 (PDB code 1U8C) were aligned to the template (gray) of the β2 fragment (PDB code 2P28) before homology modeling. On the right, the final homology model (red) was compared to the template. C. Targeting the extended β3 model (red) to the bent β3 structure (gray) by TMD simulation. Snapshots were taken at indicated times. D. The final bent β3 model (red) in the bent αVβ3 structure (αV, yellow; β3, gray). E. RMSD relative to the bent structure for all heavy atoms of the PSI, hybrid, and EGF3 domains during the TMD simulation. F. Comparison of the DOPE scores of the homology model and the template. (5.24 MB TIF) [file pcbi.1001086.s008.tif]
