# Supplementary material for: Molecular Dynamics Simulations of Forced Unbending of Integrin αVβ3
Source: PLoS Comput Biol. 2011 Feb 17;7(2):e1001086. doi: 10.1371/journal.pcbi.1001086 (PMC3040657; doi:10.1371/journal.pcbi.1001086)
Supplement: Table S1 — Summary of MD simulations for integrin αVβ3. (0.04 MB DOC) [file pcbi.1001086.s009.doc]

**Table S1. Summary of MD Simulations for Integrin α_v_β_3_**

| **Structure Name** | **U1** | **U2** | **L1** | **L2** |
| --- | --- | --- | --- | --- |
| **Structure Source** | 1U8C + modeled EGF1/2 domains | 3IJE | 1L5G + PSI domain from 1U8C + modeled EGF1/2 domains | 1L5G + PSI and EGF1/2 domains from 3IJE |
| **Protein residue range** | α_v_: 1-838 & 868-956  β_3_: 1-690 | α_v_: 1-838 & 868-967  β_3_: 1-695 | α_v_: 1-838 & 868-956  β_3_: 1-690 | α_v_: 1-838 & 868-956  β_3_: 1-690 |
| **Metal ions in protein** | 6 Ca^2+^ | 6 Ca^2+^ | 8 Mg^2+^ | 8 Mg^2+^ |
| **Ligand** | No | No | Cyclic RGD-pentapeptide | Cyclic RGD-pentapeptide |
| **Equilibration water box size** | 154×145×127 Å^3^ | 157×150×129 Å^3^ | 158×146×129 Å^3^ | 167×148×124 Å^3^ |
| **Other ions in the equilibration water box** | 224 Na^+^, 187 Cl^-^ | 241 Na^+^, 203 Cl^-^ | 224 Na^+^, 187 Cl^-^ | 241 Na^+^, 208 Cl^-^ |
| **Number of atoms for equilibration** | 244,479 | 262,033 | 256,869 | 264,434 |
| **Equilibration time** | 40 ns | 50 ns | 40 ns | 50 ns |
| **Enlarged water box size for production simulations** | 163×327×180 Å^3^ | 166×330×182 Å^3^ | 171×330×170 Å^3^ | 176×336×175 Å^3^ |
| **Other ions in the enlarged water box** | 636 Na^+^, 599 Cl^-^ | 839 Na^+^, 801 Cl^-^ | 412 Na^+^, 379 Cl^-^ | 870 Na^+^, 837 Cl^-^ |
| **Number of atoms for production simulations** | 848,486 | 902,370 | 871,271 | 937,596 |
| **SMD simulations** | 8 constant-velocity runs totaling ~106 ns & 5 constant-force runs totaling ~170 ns | 1 constant-velocity run for 10 ns & 1 constant-force run for ~17 ns | 2 constant-velocity runs totaling ~18 ns | 1 constant-velocity run for 10 ns |
| **Free MD simulations** | 4 runs totaling ~94 ns | N/A | 1 run for ~22 ns | 1 run for ~21 ns |
