# Supplementary material for: Molecular Dynamics Simulations of Forced Unbending of Integrin αVβ3
Source: PLoS Comput Biol. 2011 Feb 17;7(2):e1001086. doi: 10.1371/journal.pcbi.1001086 (PMC3040657; doi:10.1371/journal.pcbi.1001086)
Supplement: Text S1 — Building complete ectodomain models of unliganded and liganded integrin αVβ3. (0.06 MB DOC) [file pcbi.1001086.s010.doc]

**Supplemental Text S1: Building complete ectodomain models of unliganded and liganded integrin α_V_β_3_**

The first crystal structure of an unliganded integrin α_V_β_3_ ectodomain (PDB code 1U8C [1]) did not resolve the α_V_ EE’ loop (residue 839-867), the β_3_ PSI-hybrid linker (residue 51-53), and the β_3_ EGF1 and EGF2 domains (residue 435-522). The EE’ loop contains the proteolytic site after Arg860 that generates the heavy and light chains in matured α_V_ [2]. Because this loop is flexible and does not interact other domains, we left it out in our simulations. To add the missing EGF1 and EGF2 domains, we used the crystal structure of a β_2_ fragment (PDB code 2P28 [3]) as a template because β_2_ and β_3_ have ~41% sequence identity and ~54% similarity in the region containing the PSI, EGF1, and EGF2 domains (Fig. S8A). The hybrid and EGF3 domains in the 1U8C structure were first aligned to the corresponding domains in the 2P28 structure (Fig. S8B). MODELLER [4] was used to build the PSI, EGF1, and EGF2 domains with the hybrid and EGF3 domains fixed. PSI was taken into account to avoid clashes with EGF1 and to assure a long-distance disulfide bond with EGF1 (Cys13-Cys435). The β_3_ fragment model so obtained includes the PSI, hybrid, EGF1, EGF2, and EGF3 domains, which had similar quality as the β_2_ template according to the discrete optimized protein energy (DOPE) scores [5] (Fig. S8F).

Because the β_2_ template is in an extended conformation, our initial β_3_ model was also extended. To fit this β_3_ model into the bent α_V_β_3_ structure, we performed a TMD simulation without solvation using AMBER8 [6]. The extended model was first equilibrated for 1 ns (Fig. S8E). The hybrid domain in the β_3_ model was then forced to move towards the hybrid domain of the bent α_V_β_3_ with a force constant of 0.01 kcal mol^-1^ Å^-2^ with the EGF3 domain constrained. In the first ns of the bending simulation, the β_3_ model gradually bent over (Fig. S8C) and the RMSD relative to the bent structure for all the heavy atoms of the PSI, hybrid, and EGF3 domains decreased from ~100 Å to ~9 Å. The PSI, hybrid, and EGF3 domains were next forced to move towards the corresponding domains in the bent α_V_β_3_ with a force constant of 0.1 kcal mol^-1^ Å^-2^. Finally, the force constant was increased to 1 kcal mol^-1^ Å^-2^, yielding the final RMSD of ~1 Å. The bent β_3_ model so obtained had no major conflicts with the α_V_ subunit in the 1U8C structure (Fig. S8D). The PSI-hybrid linker (residue 51-53) and the EGF1 and EGF2 domains (residue 435-522) from the bent β_3_ model were added to the original 1U8C structure to generate a complete model of the unliganded integrin α_V_β_3_ ectodomain (i.e. U1).

The crystal structure of an integrin α_V_β_3_ ectodomain complexed with a cyclic-RGD ligand (PDB code 1L5G [7]) did not resolve the α_V_ EE’ loop (residue 839-867), the β_3_ PSI domain (residue 1-54), and the β_3_ EGF1 and EGF2 domains (residue 435-531). Because the 1L5G structure is nearly identical to the 1U8C structure except for regions near the ligand-binding site at the βA domain, we simply added the PSI domain (residue 1-56) and the EGF1, EGF2, and part of EGF3 domains (residue 435-548) from the bent β_3_ model to the 1L5G structure to obtain a complete model for the liganded integrin α_V_β_3_ ectodomain (i.e. L1).

References

1. Xiong JP, Stehle T, Goodman SL, Arnaout MA (2004) A novel adaptation of the integrin PSI domain revealed from its crystal structure. J Biol Chem 279: 40252-40254.

2. Suzuki S, Argraves WS, Arai H, Languino LR, Pierschbacher MD, et al. (1987) Amino acid sequence of the vitronectin receptor alpha subunit and comparative expression of adhesion receptor mRNAs. J Biol Chem 262: 14080-14085.

3. Shi M, Foo SY, Tan SM, Mitchell EP, Law SK, et al. (2007) A structural hypothesis for the transition between bent and extended conformations of the leukocyte β2 integrins. J Biol Chem 282: 30198-30206.

4. Sali A, Blundell TL (1993) Comparative protein modelling by satisfaction of spatial restraints. J Mol Biol 234: 779-815.

5. Shen MY, Sali A (2006) Statistical potential for assessment and prediction of protein structures. Protein Sci 15: 2507-2524.

6. Case DA, Darden TA, Cheatham TE, III, Simmerling CL, Wang J, et al. (2004) AMBER 8. University of California, San Francisco.

7. Xiong JP, Stehle T, Zhang R, Joachimiak A, Frech M, et al. (2002) Crystal structure of the extracellular segment of integrin α_V_β_3_ in complex with an Arg-Gly-Asp ligand. Science 296: 151-155.
